# Graphene Incorporated Electrospun Nanofiber for Electrochemical Sensing and Biomedical Applications: A Critical Review

**DOI:** 10.3390/s22228661

**Published:** 2022-11-09

**Authors:** Muzafar A. Kanjwal, Amal Al Ghaferi

**Affiliations:** Mechanical Engineering Department, Khalifa University, Abu Dhabi P.O. Box 127788, United Arab Emirates

**Keywords:** electrospinning, electrospun nanofibers, graphene, graphene oxide, electrochemical biosensors, biomedical applications, drug delivery, tissue engineering, wound healing, medical devices

## Abstract

The extraordinary material graphene arrived in the fields of engineering and science to instigate a material revolution in 2004. Graphene has promptly risen as the super star due to its outstanding properties. Graphene is an allotrope of carbon and is made up of sp^2^-bonded carbon atoms placed in a two-dimensional honeycomb lattice. Graphite consists of stacked layers of graphene. Due to the distinctive structural features as well as excellent physico-chemical and electrical conductivity, graphene allows remarkable improvement in the performance of electrospun nanofibers (NFs), which results in the enhancement of promising applications in NF-based sensor and biomedical technologies. Electrospinning is an easy, economical, and versatile technology depending on electrostatic repulsion between the surface charges to generate fibers from the extensive list of polymeric and ceramic materials with diameters down to a few nanometers. NFs have emerged as important and attractive platform with outstanding properties for biosensing and biomedical applications, because of their excellent functional features, that include high porosity, high surface area to volume ratio, high catalytic and charge transfer, much better electrical conductivity, controllable nanofiber mat configuration, biocompatibility, and bioresorbability. The inclusion of graphene nanomaterials (GNMs) into NFs is highly desirable. Pre-processing techniques and post-processing techniques to incorporate GNMs into electrospun polymer NFs are precisely discussed. The accomplishment and the utilization of NFs containing GNMs in the electrochemical biosensing pathway for the detection of a broad range biological analytes are discussed. Graphene oxide (GO) has great importance and potential in the biomedical field and can imitate the composition of the extracellular matrix. The oxygen-rich GO is hydrophilic in nature and easily disperses in water, and assists in cell growth, drug delivery, and antimicrobial properties of electrospun nanofiber matrices. NFs containing GO for tissue engineering, drug and gene delivery, wound healing applications, and medical equipment are discussed. NFs containing GO have importance in biomedical applications, which include engineered cardiac patches, instrument coatings, and triboelectric nanogenerators (TENGs) for motion sensing applications. This review deals with graphene-based nanomaterials (GNMs) such as GO incorporated electrospun polymeric NFs for biosensing and biomedical applications, that can bridge the gap between the laboratory facility and industry.

## 1. Introduction

**Figure 1:**
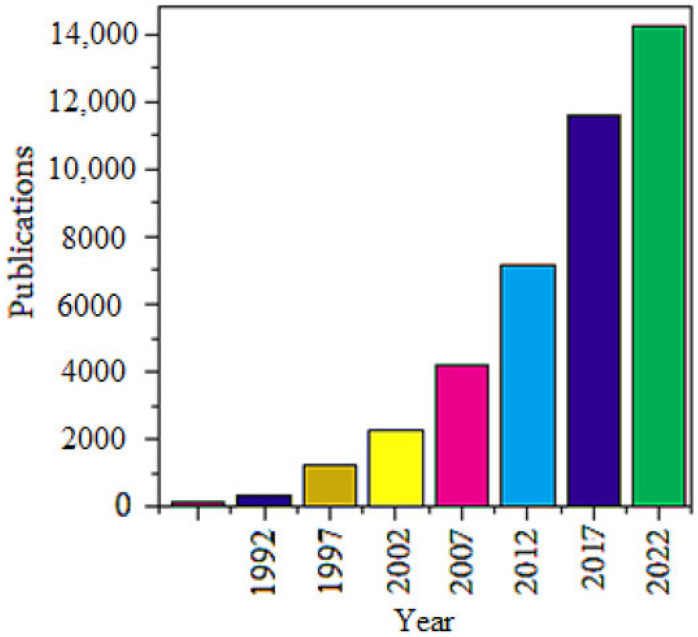
Publication trend of graphene from 1990–2022 Reprinted with permission from Ref. [35]. Copyright 2022, MDPI.

Graphene is undoubtedly one of the most outstanding carbon-based nanomaterials [1], and displays an atomic layered sheet composed of sp^2^-bonded carbon atoms, which can be produced by a top-down approach (e.g., mechanical, electrochemical, or chemical exfoliation of graphite) or by a bottom-up approach (e.g., chemical vapor deposition and chemical synthesis) [2,3,4]. In relation with other carbon allotropes, graphite, fullerenes, and carbon nanotubes, graphene presents numerous unique chemical and physical features. Due to its honeycomb lattice structure having two carbon atoms per unit cell, a linear dispersion of the energy spectrum exists, because of the close association of the valence and conduction bands and the Brillouin zone corners [5]. Consequently, charge carriers in graphene act as no-mass relativistic particles or Dirac fermions. Additionally, a powerful ambipolar electric field effect together with quick movement of charge carriers was noticed [6]. Due to the delocalization of the out-of-plane π bonds originating from the sp^2^ hybridization carbon atoms, a remarkable carrier mobility of ~200,000 cm^2^ V^−1^ s^−1^ has been realized for suspended graphene [7,8] and ~500,000 cm^2^ V^−1^ s^−1^ for a graphene established field effect transistor (FET) [9,10,11,12]. One more significant electronic feature of graphene is the exceptional fractional quantum Hall effect for charge carriers at room temperature [5,13]. Furthermore, monolayer graphene is extremely transparent when the visible light is incident on it (~2.3% absorption) [14]. Graphene has an excellent mechanical property, having a Young’s modulus of ~1.1 TPa [15]. Additionally, graphene is highly thermally conductive, revealing a thermal conductivity of ~5000 Wm K^−1^ and great surface area (2630 m^2^ g^−1^) [11,12]. Due to these distinctive chemical and physical characteristics and the distinctive biocompatibility, graphene has gained substantial attention in industry as well as scientific circles for several promising applications such as biosensors, biotechnology [16,17], clinical diagnosis [18], antibiotics and antivirals [19,20,21,22,23,24], targeted and photothermal therapy [25,26,27,28], drug distribution [29,30,31], and electric stimulants [32]. To understand the importance of graphene, we have observed the publication progress trend within scientific circles using the Scopus database (since the year 1990). The research articles that contain the word “graphene” in the title are calculated in the time stretch of 1990−2022. Figure 1 shows the growth trend of publications during these years. This observation distinctly displays the universal importance of graphene and the tremendous interest of researchers worldwide [33,34].

The group members of graphene-based nanomaterials (GNMs) consist of single and multilayer graphene, graphene oxide (GO), reduced graphene oxide (RGO), graphene oxide quantum dots (GOQDs), and graphene quantum dots (GQDs) [36,37,38]. Every group member of GNMs possesses distinct physico-chemical characteristics, that include surface chemistry, structure, composition, electrical conductivity, and mechanical strength, that are essential for a large number of operations [39,40,41,42]. Hence, GNM-based materials have a promising prospect and offer great new possibilities, not only in the energy sector [43,44,45,46], electronics [47], and environmental applications [48,49,50,51], but also in various biomedical operations, which include biosensors, noninvasive processes such as bioimaging, gene transfer therapy, drug delivery, regenerative medicine, and stem cells [52,53].

In the recent past, nanocomposites consisting of nanomaterials and polymer matrices have captured substantial interest in the area of contemporary materials science because of their outstanding thermal and chemical properties, electrical conductivity, and mechanical characteristics that can be realized at comparably less filler loading [54]. The increased performance is mostly assigned to extraordinary aspect ratio (100–1000 nm) fillers, which result in low-mass nanocomposites with tunable and diversified features which render them promising applicants for various new and exciting applications such as disease diagnosis and tissue regeneration [55,56], promoting cell proliferation [55], and identification of microorganisms and present unprecedented methods for biosensing applications. Specifically, nanocomposites consisting of GNM-based materials with polymeric materials or nanoparticles, such as metals, nanoscale hollow tubes composed of carbon atoms (CNTs), and graphene quantum dots, can perform a significant part in producing novel biosensors and promoting biomedical applications with improved efficiency [54,57,58].

In spite of promising prospects of GNMs and polymeric nanocomposites, common nanocomposite fabrication approaches such as the solvent processing method, in situ polymerization technique, and the allied processing methods experience many concerns that include the collection and accumulation of graphene in the polymeric matrices, the decrease in the electrical conductivity and mechanical strength of GNMs due to the resistant polymeric matrix, and inferior distribution of GNM nanofillers. The accumulation of graphene is because of its powerful intermolecular π–π relationships and van der Waals forces which cause inferior distribution in the polymeric matrices [59,60]. To address these issues, electrospinning offers a simple and constructive method of integrating GNMs [61,62], such as GO with good aspect ratio, into the polymeric solution, thus solving the difficulties of accumulation, by conversion of the polymeric matrices into NFs rather continuous GO sheets, thus promoting much better distribution of the exfoliated GO [63]. Moreover, characteristics such as porousness, elastic nature, hydrophobicity, mechanical properties, percolation threshold, and electrical conductivity can also be regulated by the precise control of the nanofiller dimensions, electrospinning, and solution variables [64]. GNM–polymer-based nanocomposites fabricated by the electrospinning technique have properties such as the benefits of polymers including light weight, elasticity, and malleability, as well as functional properties of GNMs that include higher mechanical strength, heat resistance, and electrochemical conductivity features [65]. Moreover, the functionalization and the distribution of GNM-based nanocomposites can be enhanced by integrating different media that include valuable metals, gold nanomaterial, basic metal oxides, minerals such as hydroxyapatite, and carbon nanomaterials that can be added either in the course of the electrospinning process or during post-processing techniques such as galvanic technology including wet chemical treatment [66]. The electrospun GNM composites, due to their excellent features, synergism, novel structures, composition, and the outstanding charge and mass transfer, are promising applicants to develop the latest technology and influence the production and commercialization of exceptionally miniature and advanced biosensors and essential wearable devices for rapid diagnostics and identification in biomedical applications [67,68,69].

Due to the absence of extensive reviews on electrospun GNMs for biosensing and biomedical platforms, this review article addresses the importance of electrospun GNM nanocomposites for fabricating biosensors and their role in biomedical applications with high sensitivity and specificity and lower limits of quantification. In addition, incorporation of GNMs into NFs in the course of the electrospinning technique utilizing the pre-processing approach or post-electrospinning process as surface treatment and functionalization utilizing post-treatment techniques is discussed. Furthermore, the characteristics of electrospun GNM-based nanomaterials and their importance in biosensing and biomedical applications are precisely discussed.

Graphene’s characterization is an interesting feature of the study and investigation of graphene. Characterizations include the examination of graphene morphological attributes, characteristics, deformity, and substrates and are based on spectroscopic and microscopic investigations [70,71], Raman spectroscopy [72], scanning electron microscopy (SEM), transmission electron microscopy (TEM), X-ray diffraction (XRD), ultraviolet–visible spectroscopy (UV–Vis), and atomic force microscopy (AFM) which are some of the important characterization techniques utilized [73].

## 2. Fabrication of GNM-Based Materials

**Figure 2:**
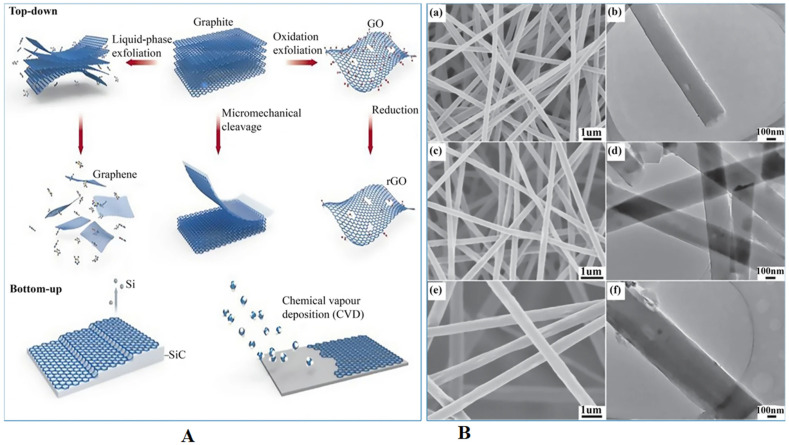
(**A**) Major fabrication methods of graphene: Top-down and bottom-up fabrication methods. Principal top-down methods include liquid-phase exfoliation and micromechanical cleavage of graphite. An additional method involves the exfoliation of initially oxidized graphite, leading to GO, which is chemically and/or thermally reduced to graphene. The bottom-up fabrication of graphene is usually performed by epitaxial growth on SiC or chemical vapor deposition, typically on Cu using small molecules, such as methane, as precursors. Reproduced with permission from [104]. Copyright Springer Nature 2020. (**B**) SEM (**a**,**c**,**e**) images and TEM images (**b**,**d**,**f**) of NFs (**a**,**b**), NFs-rGO-5 (**c**,**d**), and NFs-rGO-10 (**e**,**f**) with different magnifications. Reproduced with permission from [104]. Copyright 2019 Wiley.

GNMs such as graphene, GO, rGO, and GQDs can be fabricated employing two types of production techniques: (i) top-down and (ii) bottom-up approaches (Figure 2) [74]. The former depends on exfoliation of graphite layers by using different physical or chemical methods and heat treatment to fabricate graphene and it involves mechanical exfoliation [75], molecular assembly [76], conductive polymeric materials [77], and hydrophilic polymers [78]. The bottom-up approaches involve chemical vapor deposition (CVD) and chemical synthesis fabrication approaches [2,3]. The electrochemistry of GNMs relies on several parameters such as defects per unit, functional groups, number of layers, dimension of graphene sheets, and nature of the doping agent used [79,80,81,82,83,84]. CVD is a technique utilized to accumulate graphene layers of superior grade, fine aromatic arrangement with fewer intrinsic defects, dense components, higher reactivity, specific conductance, and high elastic nature which render it an extremely promising and potential candidate for sensors [85] and biological applications to identify microorganisms [86,87,88,89,90]. In ambient conditions, single-layer graphene (SLG) has excellent electron conductivity of around 250,000 cm^2^/(Vs) [91], therefore facilitating its applications in photonic and electronic devices. Generally, the CVD technique is the fast and effective approach to grow graphene flakes on various transition metal surfaces that include germanium [92,93], nickel [94,95], copper [96,97], and rhodium [98,99]. Subsequently, a top-down approach such as Hummer’s method is employed to produce excellent grade oxidized graphene sheets of varying size, better solution processing, various oxygen-containing functionalities, and distinct graphene layers [100]. GO is a useful and promising material form of graphene [101] consisting of sp^2−^- and sp^3−^-bonded carbon atoms, a large number of carboxylic acid groups, oxiranes, and hydroxyl groups, mostly on the boundary and deformity of the nanosheet, therefore resulting in the formation of a sheet-shaped amphiphilic colloid structure [102]. GO, because of the significant number of residual sp^2^ carbon atoms and a strong affinity for water groups, can form reliable suspensions in water and many other polar solvents and can establish π–π associations with aromatic compounds [103]. The GO functional groups containing oxygen enhance its distribution in polymer matrix and decrease the accumulation and phase separation. GO can interact with several nanomaterials based on its amphiphilic features and can also perform as a surface active agent [102]. RGO can be procured by reduction (physically or chemically) by heat and radiation approaches, that are economical methods to produce graphene layers having excellent conductivity. rGO has more stabilized physico-chemical characteristics such as surface functional groups, electrical conductivity, mechanical properties, solvent distribution, optical properties, and thermal efficiency in relation to graphene and GO [103]. Owing to these features, rGO nanosheets are promising applicants for advanced and future-generation electronics, biosensors, and biomedical fields. GQDs are graphene and GO nanoparticles with a size less than 20 nm. Due to their outstanding characteristics including less toxicity, luminescence, chemical stability, and prominent quantum confinement, GQDs are regarded as a unique nanomaterial for biological, energy sector, and environmental applications. GQDs are fabricated through a top-down method such as chopping of graphene or GO nanomaterials [104].

### 2.1. GNM-Loaded NFs

Electrospinning technology has captured significant interest in the last few decades, as it yields a versatile and unique tool for producing nano/micromembranes in a continuous and controlled process and with uniform pore size distribution, and the fiber diameters can be regulated from nanometers to sub-micron size [105,106,107,108,109]. In the recent past, NFs having diameters in the sub-nanometer (less than 1 nm) range have been reported [110,111]. Even though many alternative techniques are available for the nanofiber fabrication, that include self-assembly soft lithography, melt film fibrillation, centrifugal spinning, and drawing, electrospinning is the most widely used technology, because of its simplicity, low cost, and versatility with excellent potential to produce great quality NFs with multiple applications and well-regulated morphologies [112,113,114]. Electrospinning technology has been effectively employed to fabricate NFs from a broad spectrum of materials, such as organic or inorganic polymers, ceramics, a range of metals, carbon materials such as graphene and carbon nanotubes, micromolecules, and biomolecules in addition to microorganisms such as bacteria, viruses, and fungi [107,115,116]. The integration of GNMs into NFs allows substantial improvement in biosensing platforms by enhancing the response features of the transducer or by facilitating the immobilizing of matrix for a biosensor [117]. GNMs can be integrated into the NFs by employing two fundamental approaches: (i) pre-processing techniques (blending process and in situ synthesis) and (ii) post-processing techniques (vacuum slurry dip coating, ultrasonication, plasma injection, wet chemistry, and radiation method) [118,119].

### 2.2. Engineering Electrospun GNM Composites Using Pre-Processing Techniques

Incorporating GNMs into the polymeric solution for electrospinning is an easy and efficient approach to produce electrospun composites containing GNMs for several exciting platforms that include sensors and biological applications [61]. The stability of GNMs is critical to establish the extended storage and adsorption and outstanding recyclability properties of GNM-based electrospun nanofiber composites for biosensors and biomedical applications. Here, GNMs play an important role in electron transfer, whereas polymers work as precise adsorptive material for biological assay, therefore GNMs and polymers work in synergy for biosensor and biological applications. NFs containing GNMs create links among biomolecules and cell signaling and therefore play an integral part in the construction of sensor and conduction segments of biosensors. Great distribution and proper dispersal of GNMs into the polymeric matrix allow the production of electrospun nanofiber composites with a high degree of functionality, unique hierarchical structures, higher surface to volume ratio and controlled porosity, outstanding physico-chemical properties, electrical conductivity, and electrochemical characteristics, rendering them as outstanding platforms for rapid diagnostics and micro total analysis systems [117]. The two well-known methods to ensure homogenous dispersion of GNMs into matrices of polymeric NFs are (i) direct blending and (ii) in situ synthesis during electrospinning.

### 2.3. Direct Blending of GNMs in Electrospun Polymeric NFs

Direct blending of GNMs into polymeric matrix solution is the primary and simple way to produce GNM incorporated nanofiber composites. Employing the direct blending method may reduce the surface energy of GNMs which results in crosslinking of test GNMs and polymeric matrix. Regarding the biosensor and biomedical applications, the uniform distribution of GNMs into the polymeric matrix is a critical feature for enhancing the sensitivity and limit of quantitation for rapid diagnostics and lab-on-chip devices. Consequently, other ways to ensure and enhance the uniform distribution of GNMs into the polymeric matrix should be explored.

### 2.4. Uniform Distribution of GNMs Utilizing External Forces

The principal issue in producing NFs containing GNMs is their high free energy and huge surface to volume ratio, that results in agglomeration of GNMs and therefore undermines their eventual performances in biological and biosensing fields [57]. The agglomeration of GNMs into polymeric matrix could be due to their local association with the polymeric molecules and the random overlapping of an interphase of adjoining GNMs or polymeric materials used. Hence, the nonhomogeneous distribution of GNMs into polymer matrix at the nanoscale may affect the efficiency of the electrospinning technique and solutions. The decreasing GNM loading potential may have a negative impact on the general material properties and applications. To address these problems, the electrospinnable solutions can be treated with an exterior force to provide uniform distribution of GNMs, including multiple attempts of blending and aggressive stirring, different ultrasonication approaches, functionalization, and surface engineering of GNMs with surface active substances to facilitate proper distribution of GNMs. Inclusion of additives helps to alleviate the large surface energy gap to obtain a significant solvability and proper nanoscale dispersion, therefore increasing and facilitating their electrospinnability. Different kinds of spacers, that include metal nanoparticles and basic metal oxides [120,121], different functional groups [122,123], and several polymers [120,124] have been inserted into graphene materials to increase the distribution and the specific surface area to yield additional adsorption sites for biosensing and biomedical applications.

### 2.5. In Situ Synthesis of GNMs into Polymeric Nanofiber Matrix

Like blending, in situ synthesis is an efficient method to distribute GNMs into the polymeric matrix to fabricate NFs containing GNMs, utilizing several approaches that include the hydrothermal method, chemical solution deposition, redox reaction, and hydrolysis reactions. Employing this method, graphene material distribution into polymeric solutions could be achieved by electrochemical, thermal, photo, and active agents to evenly disperse GNMs into polymeric matrix with dimension control and homogeneity and escaping the aggregation of graphene-based materials. One research team [125] reported a simple and single-step approach for in situ fabrication and arrangement of electrospun graphene-doped zinc oxide composite NFs. They regulated parameters such as the temperature and time in the electrospinning technique to produce aligned electrospun graphene–zinc oxide composite NFs over a gold electrode. The published approach is an economical way to detect UV and it can be applied to a range of biosensing and biomedical applications. Another research team [126] published the electrospinning in situ synthesis of graphene-doped porous copper indium disulfide/carbon composite NFs for a highly efficient counter electrode in dye-sensitized solar cells, in which graphene nanosheets attached to porous copper indium disulfide nanocrystals of 7–12 nm in diameter were overlaid and encapsulated in a carbon matrix, aligning across the nanofiber axis. Subsequently, the composite graphene NFs revealed less charge transfer resistance, huge surface to volume ratio, and outstanding electrocatalysis in relation to copper indium disulfide nanocrystals/carbon and p-copper indium disulfide nanocrystals/carbon samples.

### 2.6. Dispersal of GNMs Using Electrospinning

Electrospinning technology utilizes the electrostatic charge to overcome any disentanglement and aggregation of GNMs by enhancing their intersection area with the polymeric matrices, subsequently facilitating the formation of potential chemical bonds between GNMs and test polymers. Electrospinning delivers tangential stress transfer mechanics from the polymeric matrix to the nanometer-sized GNMs, hence increasing the distribution of GNMs and avoiding their agglomeration. In addition, in the course of the electrospinning process, the high stretching of the polymeric jet under the influence of an electric field significantly enhances the alignment of graphene materials across the nanofiber axis and incorporates them in the nanofiber nucleus, thus accomplishing extremely dispersed electrospun GNM composites. The amount of GNMs has a substantial impact on their distribution and therefore prompts the changes in rheological and physical properties that include the specific conductance and viscosity and the dimension of the NFs. For example, increasing the content of GNMs results in higher viscosity and eventually the formation of NFs of larger diameters [127]. However, increasing the content of GNMs increases the specific conductance, which facilitates the stretching of fine NFs [127]. Due to these contrasting actions, some reports have shown fluctuating nanofiber diameters as the amount of the GNMs is enhanced [127,128]. One research team [129] reported a double approach consisting of electrospinning and electrospraying to address the problem of blending and distribution of polyacrylonitrile (PAN) and GO in an identical solvent. Another research team [106] reported the production of free-standing nitrogen-doped graphene–carbon nanofiber composite mats by electrospinning synthesis and application. These NFs exhibited high conductivity, large specific surface area, and chemical durability and therefore had the great potential to be employed for chemical sensing, separation, and biomedical applications. Figure 2b shows the scanning electron microscope (SEM) results for the developed PAN-GO electrospun nanofiber mats.

### 2.7. Fabrication of GNM NF Composites Using Post-Processing Approaches

**Figure 3:**
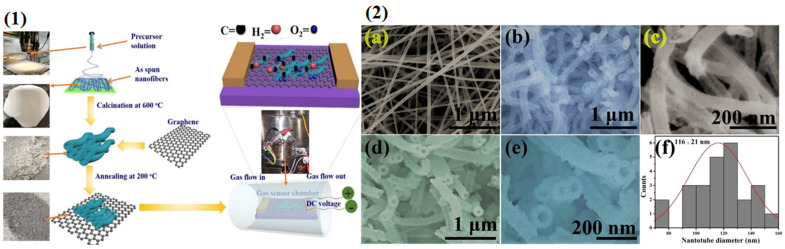
(**1**): Pristine and GO-SnO_2_ NT preparation and gas sensor mechanism and (**2**) SEM images of (**a**) as-prepared Sn^+^poly (vinyl pyrrolidone) (PVP) NFs, (**b**,**c**) pristine SnO_2_, and (**d**,**e**) GO incorporated SnO_2_ NTs, (**f**) histogram of GO-SnO_2_ NT diameters. Reproduced with permission from [135]. Copyright 2019 Elsevier.

Even though the direct blending of GNMs into NFs is the easiest and most constructive approach, one of the principal issues of blending GNMs into the polymeric matrix is that GNM nanofiber composites exhibit comparatively low electrical conductivity because the conductivity of graphene-based materials can be deformed within insulating polymeric materials due to various external factors. Another method is to impregnate graphene-based materials onto the surface of NFs after the electrospinning process by applying a surface modification procedure as a post-processing approach. This method avoids the issues related to blending of GNMs into the polymeric matrices such as aggregation and low electrical conductivity and provides an efficient platform to develop the sensing and biological features of GNM electrospun nanocomposites. Generally, post-processing procedures utilize the impregnation or coating of graphene-based materials on the surface of the test NFs by employing physico-chemical procedures to modify the surface of the electrospun NFs by imparting them new properties (e.g., plasma activation, increasing surface specific conductance) [130]. These procedures have a significant impact on abundant reactive zones for molecular immobilization while analyzing the surface characteristics of the NFs that are directly proportional to the chemical structure of the electrospinning solution and the surface design of the NFs [131]. This strategy is significantly easy and smooth to execute and is inexpensive at an industrial level compared to direct blending of polymeric material with graphene-based materials. Moreover, the deposition of GNMs should be carried out in such a way that NFs contain the maximum loading of GNMs and have maximum possible prospects of interaction with biosamples which is essential for biosensors and biomedical applications [132]. The integrating of electrospun NFs with GNMs for biosensing and biomedical applications includes procedures such as physisorption and surface coating, surface modification by graft polymerization, plasma treatments, chemical doping, and wet chemical analysis. Physical dip coating is the most straightforward and easiest out of these approaches to provide electrospun NFs with GNM active zones for selected reactions. This procedure is dependent on the relationship of the molecular sensitive probe with the electrospun NFs involving weak forces such as van der Waals forces, hydrophobic interactions, Coulomb interactions, and electrostatic dipole–dipole interactions [133]. Nevertheless, the effectiveness and stability of biomolecular immobilization in this approach are comparatively inadequate [134]. To address this issue, the plasma treatment procedure increases performance and effectiveness of physisorption onto the hydrophobic NFs by generating a more hydrophilic surface, thus increasing biological molecular affinity, due to the abundant presence of carboxyl groups on the hydrophilic surface. The layer-by-layer approach provides a way to alter the surface properties of NFs by employing Coulombic force of attraction to influence the physical, chemical, biological, and mechanical features of aggregated polymer electrolyte multilayers, enabling control at the nanometer level over arrangement and design. Atomic chemical doping is an efficient way to acquire elemental alteration of GNMs to increase their electrochemical features [135]. One research team [136] reported the fabrication of GO incorporated SnO_2_ nanotubes in which the SnO_2_ nanotubes were synthesized by a single-step electrospinning technique and GO was doped into SnO_2_ nanotubes (GO-SnO_2_ nanotubes) by dipping SnO_2_ nanotubes in a GO solution with proper heat treatment as shown in Figure 3(1). They started off with calcination of the prepared electrospun SnO_2_ nanotube fibers at 600 °C for 2 h to eliminate polymer residues and the organic solvents and the oxidation of the inorganic precursors took place and resulted in the formation of SnO_2_ nanomaterials. Subsequently, 0.03 g of pure SnO_2_ nanotube packs was immersed into 1 mL of GO solution containing DI water and desiccated in atmospheric oxygen for multiple hours. Moreover, GO-loaded SnO_2_ nanotubes were eventually collected after heat treatment at 200 °C. The alteration of SnO_2_ nanotubes by GO exhibits much better sensing abilities (including quick response time) due to the high number of interfacial relationships among the GO and the SnO_2_ nanotubes and the results are displayed as SEM images in Figure 3(2).

### 2.8. Characteristics of NF-Loaded GNMs

**Figure 4:**
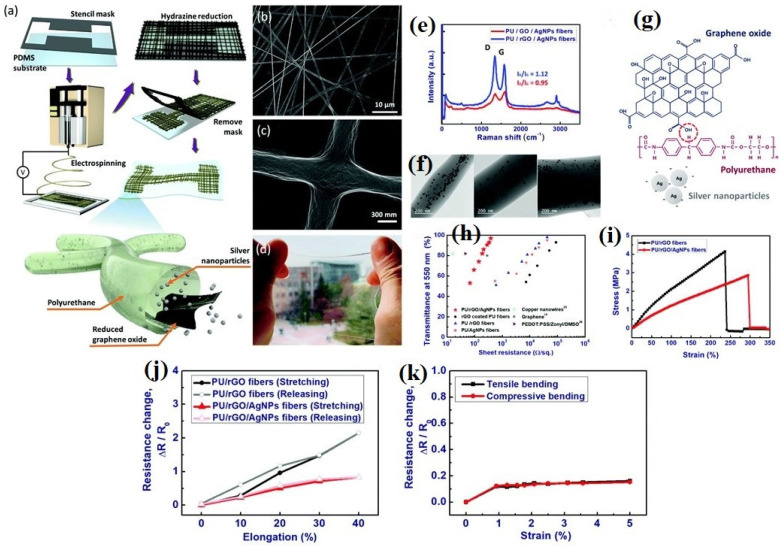
(**a**) Technological flow chart of the patterned STNNE. (**b**) FESEM image of the networked NFs. (**c**) FESEM image of the intersections of the NFs. (**d**) Optical photographs of the stretchable and transparent networked NF film. Dispersion of PU/rGO/AgNPs in NFs. (**e**) Raman spectra of PU/GO/AgNP nanofiber and PU/rGO/AgNP nanofiber samples with a GO:AgNP loading ratio of 1:1.25. (**f**) TEM images of NFs with diameters of ~290, ~484, and~933 nm. (**g**) Schematics of the functional groups on GO, chemical structure of polyurethane, and negative surface charges of AgNPs. GO nanosheets can be hydrogen-bonded to the PU matrix by the functional moieties of the carboxyl and hydroxyl groups. (**h**) Optical transmittance sheet resistance of the networked NFs for different types of NFs: rGO-coated PU, PU/rGO, PU/AgNP, and PU/rGO/AgNP NFs with that of copper nanowires, PEDOT: PSS/Zonyl/DMSO, and graphene. (**i**) Stress–strain curves of PU/rGO and PU/rGO/AgNP NFs. Evaluation of STNNEs under stretching conditions. (**j**) Resistance change (ΔR/R0) versus elongation of the PU/rGO and PU/rGO/AgNP nanofiber electrodes on PDMS substrates. (**k**) Resistance change (ΔR/R0) versus low strain under tensile and compressive bending of STNNEs. Reproduced with permission from [104]. Copyright 2020 Springer Nature.

**Figure 5:**
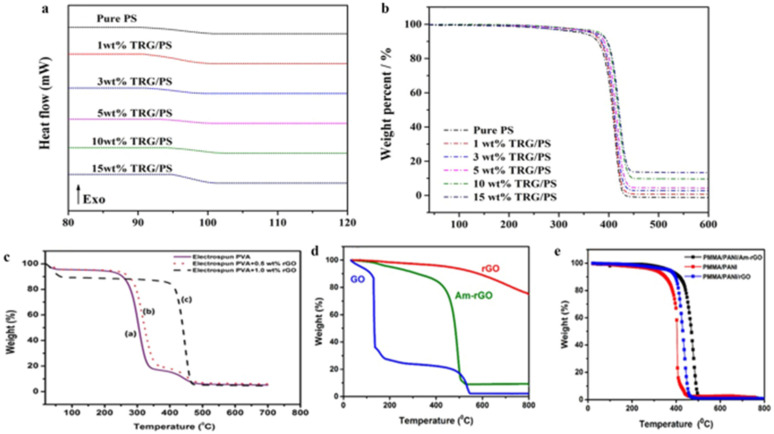
Dimensionally stable anodes (DSC) (**a**) and thermogravimetric analysis (TGA) (**b**) curves of pure PS matrix and the TRG/PS nanocomposites. Reproduced with permission from [154]. Copyright 2018 Elsevier. (**c**) TGA curves of electrospun PVA mats mixed with GO. Reproduced with permission from [155]. Copyright 2019 American Scientific Publishers. (**d**) TGA curves of rGO, rGO, and AM-rGO. (**e**) TGA curves of electrospun PMMA/PANI/AM-rGO, PMMA/PANI/rGO, and PMMA/PANI NFs. As shown in (**e**), the thermal degradation temperature of PMMA/PANI/Am-rGO NFs increased to~441 °C, a magnitude higher than that of the PMMA/PANI samples at~348 °C. Both (**d**,**e**) are reproduced with permission from [156]. Copyright 2017 MDPI.

NFs are differentiated by their outstanding functional characteristics which include a large specific surface area, controlled diameter, higher aspect ratio, molecular orientation which is the extension of macromolecules and other structural units along the nanofiber axis, tuned porosity, reasonable mechanical properties, various nanofibrous morphologies, and excellent physical, chemical, and electrical characteristics [64,137,138,139,140]. Due to these diverse features, electrospun nanofibers are promising candidates for a broad range of applications such as increasing the efficiency of analytical gadgets and biomedical and biosensor applications [107]. Incorporating GNMs into NFs in the course of the electrospinning technique either during pre-processing approaches or after the electrospinning technique employing post-processing approaches endows NFs with outstanding features and morphological compositions, essential for biosensing and biomedical applications. With respect to electrochemical and adsorption qualities, the three-dimensional interrelated composition of GNMs facilitates the distribution of several kinds of macromolecules and preserves their biocatalytic activity, therefore enhancing the sensitivity and functionalities for biomedical and biosensing applications. GNMs are excellent supplements to enhance the mechanical strength and electrical conductivity of NFs. Due to their innate higher stability and strength obtained from the powerful carbon bonds and their associations with the polymeric matrix and their distribution, the inclusion of GNMs can significantly enhance the mechanical properties of the NFs [141]. There are many reports where the dispersal of GNMs into polymeric matrix enhanced the mechanical strength, electrical conductivity, and thermal characteristics of polybutylene terephthalate [142,143], poly (ethylene terephthalate) [144,145], nylon [144,145], polyimide [146,147], polyaryl sulfones [148,149], and polycarbonates [150,151]. One research team [152] reported that the integration of GO into electrospun PU NFs and pH-responsive dyes displayed a rapid response time of 7 s and enhanced the sensing ability and sensitiveness to determine pH in a vapor test. Similarly, another research team [153] reported that a stretchable and transparent nanofiber-networked electrode established NFs of polyurethane (PU)/rGO/silver nanoparticles (AgNPs) with excellent distribution as shown in Figure 4. It was established that the excellent distribution of AgNPs into the PU/rGO NFs increased the specific conductance and mechanical stretchability. Moreover, the existence of rGO and the development of fused intersections among NFs which happened in the course of the electrospinning process had a positive impact on the electrical properties of the produced stretchable and transparent nanofiber-networked electrode. The manufactured stretchable and transparent nanofiber-networked electrode was effectively established as a stretchable capacitive sensing device. Another research team [154] reported a rise in the thermal conductivities in polystyrene nanocomposites by integrating thermally reduced graphene oxide through an electrospinning and hot press technique. In particular, the incorporation of 15 wt% thermally reduced graphene oxide enhances the thermally conductive coefficient (λ) of pristine polystyrene more than three times to 0.689 W/mK, glass transition coefficients (a) around three times to 0.6545 mm^2^/s, glass transition temperature (Tg) from 90.3 to 95.0 °C, and heat-resistance index (THRI) from 184.2 to 194.3 °C. The addition of rGO to polyvinyl alcohol enhanced the thermal stability considerably as shown in Figure 5c [155]. Similarly, thermal stability of PANI increased after graphene inclusion as shown in Figure 5d,e [156].

In addition to electrospinning, another important method used to incorporate graphene into NFs is chemical vapor deposition (CVD). The CVD of graphene films includes the decomposition of a fluid at elevated temperature to deposit graphene on NFs. Evaporated Ni films on SiO_2_/Si wafers or copper foils are the best media for graphene synthesis [156,157,158,159]. The CVD method can be exploited as one of the high-efficiency fabrication approaches [160] and it has been shown that the accumulated graphene film can be shifted from the initial substrate to several other substrates [159,161]. Moreover, this fabrication approach is possibly convenient for operations where a graphene film is needed on an extensive range of substrates that cannot resist elevated temperature influence [162]. The other methods include mechanical exfoliation, etc.

### 2.9. Electrospun GNM Nanocomposites as Electrochemical Biosensors

Electrochemical biosensors are typically an analytic tool effective in converting the feedback of biological tests into current signals and consist of two segments, the biological detection segment and the transduction segment. The biological detection segment is of principal importance and is composed of a biosensing element (such as aptamer, enzyme) that helps in precise determination of the biological tests and subsequently yields current signals. The transduction segment modifies these observed signals into clear readout signals for additional evaluation and estimation.

In the recent past, several approaches have been developed to identify nitrate and nitrite in water. One of the most important methods is to use graphene-based materials. It is primarily utilized for developing electrodes for electrochemical sensors. Graphene-based electrochemical sensors have the striking properties of being economical, effective, and precise for nitrite and nitrate detection [163]. Moreover, in relation to pathophysiology, many research works have become essential to produce biosensors for early detection of disease and diagnostics, exploiting nanomaterials such as quantum dots (QDs). These QDs efficiently improve the sensor performance in relation to their duplicability and preciseness in addition to sensitivity, rendering them potential applicants for a broad range of novel biomedical operations [164]. In another report, functionalized graphene gas sensors have been reported, which have drawn a lot of research interest and importance, because of their ability for high sensitivity, outstanding selectivity, and quick identification of several gases. For instance, functionalized graphene sensors for ammonia (NH_3_) detection at ambient temperature [165]. In another study, an arginine-modified carbon interface and arginine-modified CPE (AMCPE) for the cyclic voltammetric sensing of dopamine (DA) were reported. The AMCPE exhibited outstanding results in identifying DA in blood serum specimens [166].

Integrating GNMs into NFs to manufacture electrochemical biosensors is gaining great attention globally from the scientific community primarily due to their excellent sensitivity and lower limit of detection, that depend on their electrochemical properties, oxidation–reduction reactions, electrocatalysis, and charge transfer kinetics [167]. In addition, GNMs have some other outstanding features which include larger surface area, economy, and great charge transfer potential [168]. In electrospun GNM biosensors, NFs do not play any role in detection or transduction due to the absence of active receptors and help only as the supporting material to GNMs as well as the bioreceptors. However, GNMs function as the detection and transduction segment, because of their great adsorption and precise sensitivity for target components. The homogenous dispersal and distribution of GNMs into ESNFs enhance the reactivity, accelerate the adsorption rate, and yield abundant GNM active zones and functions by immobilizing bioreceptors in electrochemical biosensing platforms and, subsequently, speed up the charge transfer rate between the analyte and the transducer part and play a critical role in maintaining their bioactivity in biosensing applications [169]. Moreover, the configuration and morphology of electrospun GNMs are essential parameters as they regulates the porosity that permits fluids to move through pores with less mass resistance, therefore enhancing the target biofluid dispersal proceeding towards the electrode and, subsequently, yielding precise and sensitive observations [170,171].

**Figure 6:**
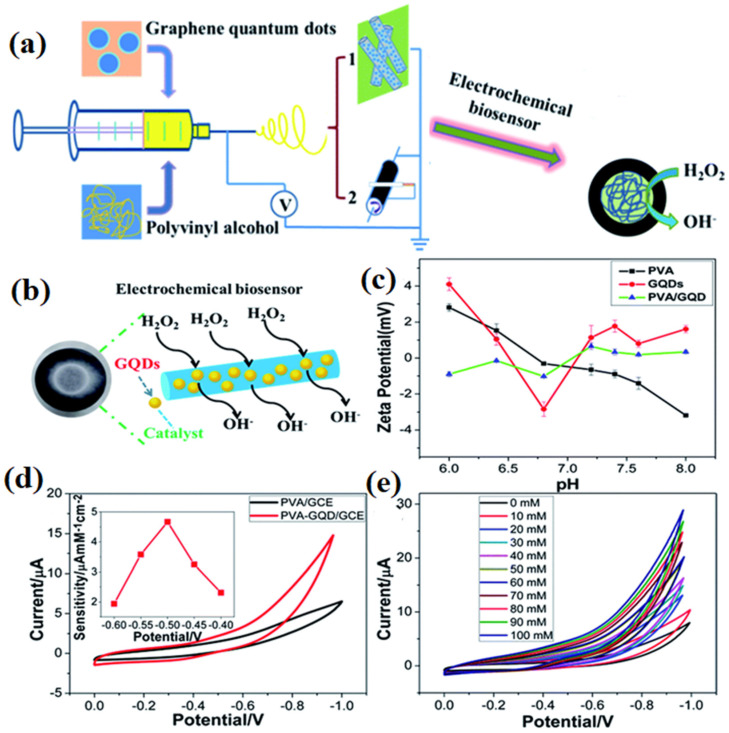
(**a**) Schematic presentation of electrospinning for depositing PVA/GQD onto GCE for electrochemical biosensing and catalyzing of H_2_O_2_, (**b**) the possible detection mechanism, (**c**) zeta potentials of GQDs, PVA, and PVA/GQD nanofibrous membranes at varied pH, (**d**) CVs of GCEs modified with PVA and PVA/GQD nanofibrous membranes, sensitivity of the biosensor at different potentials (inset), (**e**) CVs of the PVA/GQD nanofibrous membranes modified GCE 0.1 M PBS with different additions of H_2_O_2._ Reproduced with permission from [104]. Copyright 2020 Springer Nature.

Electrospinning is a simple and appropriate technology to produce NF biosensors from a broad spectrum of microporous materials (pore diameters less than 2 nm) and mesoporous materials (pore diameters between 2 nm and 50 nm) [117]. Electrospinning provides a polymeric nanofiber and regulates pore geometry, dimensions, and functionalization which helps in the generation of unique nanomaterials with excellent biosensing properties [170]. The functionalization of NFs can be achieved by integration of GNMs in the course of the electrospinning process or post-electrospinning process on the surface of NFs to increase fundamental properties such as specific conductance, electrochemical characteristics, and redox transfer, which results in increasing the rate of chemical reactions essential for producing electrochemical biosensors. Due to their high surface to volume ratio and excellent porosity, NFs can yield numerous immobilization zones and therefore can be attached to biorecognition elements by utilizing coupling chemistry permitting the intersection of biorecognition analytes and increasing the signal processing for the biomolecules under investigation. The smooth fabrication of a supersensitive, effective, and repeatable electrochemical biosensor was reported [172] for the detection of H_2_O_2_ by utilizing polyvinyl alcohol, GQDs, and electrospinning technology as shown in Figure 6. The electrospun GQD electrochemical biosensor exhibited a linear range of detection of 0.1–200 mM and a limit of quantification of 0.53 μM. The fact was established that GQDs can substitute the conventional semiconductor QDs and upgrade the electrochemical characteristics of carbon-based material. A unique perceptive and extremely selective electrochemical biosensor established on polyvinyl pyrrolidone (PVP)/chitosan (Chi)/rGO NFs for synthetic estrogen (17 α-ethinyl estradiol (EE2)) was reported by one research team [173]. Before the characterization of the NFs and before immobilization of laccase enzyme, the produced PVP/Chi/rGO NFs were processed with glutaraldehyde solution, followed by crosslinking of the PVP/Chi/rGO NFs. The crosslinking of these composite NFs is important for the activation of the amine and hydroxyl functional groups from chitosan and graphene sheets, for laccase enzyme immobilization. Molecular bonding was used for the immobilization of the laccase enzyme on the PVP/Chi/rGO NFs by amine groups of laccase enzyme and the activated functional groups from the PVP/Chi/rGO NFs. The PVP/Chi/rGO/laccase electrode was utilized for the detection of synthetic estrogen EE2. The incorporation of chitosan and PVP with rGO enhanced the electron transfer which resulted in outstanding biosensing characteristics. Figure 6a shows the fabrication of the electrochemical biosensor with respect to immobilized laccase enzyme on the FTO/PVP/Chi/rGO NFs and the cyclic voltammetry, electrochemical impedance spectroscopy (EIS), and amperometry response are displayed in Figure 6b–d, respectively. Mesoporous poly (styrene-block-methyl methacrylate) (PS-b-PMMA) NFs were reported [114] to increase the analytic efficiency of an electrochemical biosensor by utilizing the impact of porousness and surface area on the biosensing potential of PS-b-PMMA electropsun NFs. Coupling chemistry was used to functionalize the PS-b-PMMA NFs and oxidation–reduction reactions were used to investigate the existence of the carboxyl functional groups. Figure 6e displays the cyclic voltammetry outcome of the fabricated electrochemical biosensor. The fabricated electrochemical biosensor exhibited a reasonable selectivity and sensitivity enhancement by around 2.7 times, a linear range of detection of 10 fM–10 nM, and a limit of quantification of 0.37 fM.

**Figure 7:**
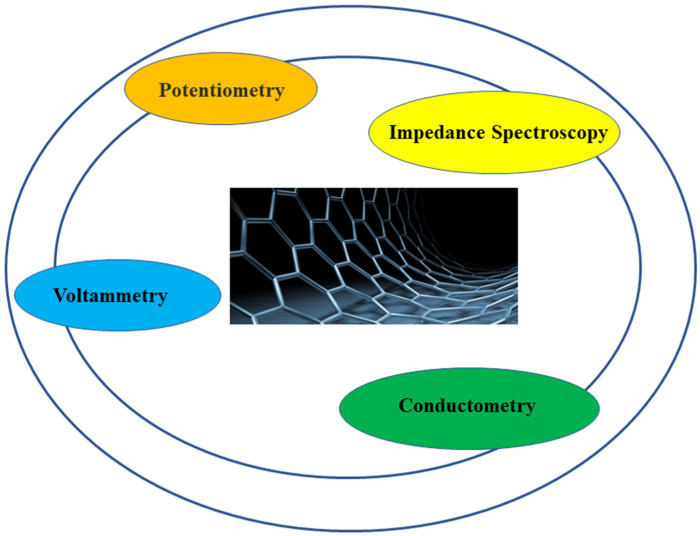
Scheme showing the applications of graphene in different sensor streams.

In another study, graphene loaded with MnCo_2_O_4_ composite NFs (GMCFs) was fabricated by electrospinning technology, followed by calcination in an Ar atmosphere. The fabricated GMCFs were exploited as an effective platform for glucose biosensing. Electrochemical investigation revealed that the fabricated biosensor displayed outstanding electrocatalytic activity in relation to glucose oxidation over a broad range of 0.005–800 mM with a lower limit of detection of 0.001 mM [174]. In another study, fabrication of an extremely sharp and selective electrochemical aptasensor utilizing electrospun cellulose acetate (CA)-doped three-dimensional (3D) graphene for ochratoxin A (OTA) evaluation was reported. The distinct electrospun CA/3D graphene surface was made functional by silane and glutaraldehyde functional groups to tether terminal linkers for incorporating OTA aptamer utilizing the layer-by-layer deposition approach. The current–voltage measurements of the aptasensor displayed a lower limit of detection of 156 fg ml^−1^ and sensitivity at 0.3 nA with a linear range of detection of 1 fg ml^−1^ to 1 ng ml^−1^ [175]. Figure 7 shows the scheme regarding the applications of the graphene in different sensor streams.

## 3. Biomedical Applications of Electrospun Graphene Oxide

### 3.1. Biomedical Prospects of GO

GO is a monolayer material and chemically composed of carbon, oxygen, and hydrogen [176]. GO is an oxidized type of graphene and is composed of several carbonyl (RCOR), carboxyl (RCOOH), hydroxyl (ROH), and epoxy functional groups [177,178]. GO is a hydrophilic material in essence and can easily form stable aqueous colloidal suspensions, that enable the development of macroscopic structures with economical operability [177,179]. GO possesses several surface defects and the core material exhibits substantial similarities to pure graphene [180]. GO is a very promising material in the biomedical field due to its favorable chemical structure. GO has important applications in smart drug/gene delivery because of its exceptional surface area [181]. The nanosheet structure of GO can safeguard biomaterials from deterioration, increase the chemical bonding between biomolecules and the core parent chain, and also enhance their distribution time [181,182]. The oxygen-rich nature, hydrophilicity, and superflexibility of GO permit excellent cell growth and expansion in biomedical engineering [183,184]. Moreover, due to striking features such as antibacterial and antimicrobial properties and drug delivery ability, GO is a very promising applicant for wound healing applications [185]. The development of new blood vessels by GO is found to be dose-dependent and is well documented [186]. GO and reduced graphene oxide (rGO) can enhance the number of reactive oxygen species (ROS) (ROS include peroxides, superoxide, hydroxyl radical, singlet oxygen, and alpha-oxygen) in organisms, thus promoting formation of new blood vessels [186]. GO, due to its innocuous nature and biocompatibility, has great potential in medical instruments [187,188,189]. However, it is also well documented that concentrations of GO above a certain optimum level may result in a reduction of biocompatibility [190,191]. The ROS generated from GO induce oxidative stress when antioxidant levels are low, such as caspase 3-mediated apoptosis (caspases are crucial mediators of programmed cell death) under the influence of adrenal pheochromocytoma PC12 cells [192,193,194]. Therefore, it is essential to combine GO with other suitable and established biomaterials to ensure increased biocompatibility. One method to achieve this is by employing electrospinning technology. It is possible to electrospin GO and different biopolymers to form GO-loaded electrospun nanofibrous composites [194]. The selected polymer should be biocompatible, biodegradable, and harmless in nature.

### 3.2. Electrospinning in Tissue Engineering

**Figure 8:**
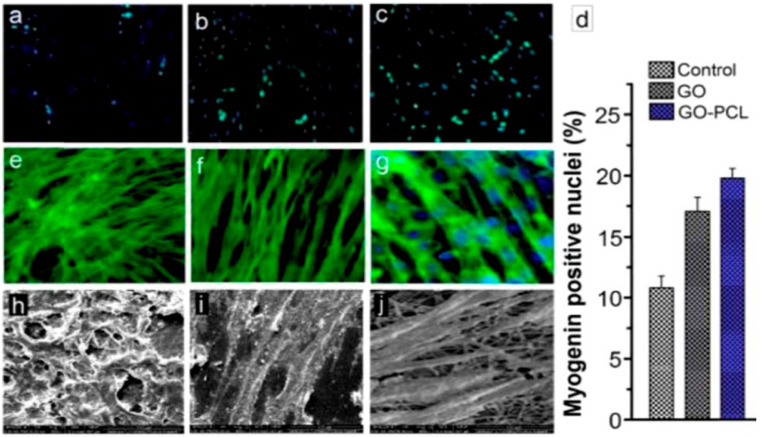
Myogenin on tissue culture plate stained using immunofluorescence taken as control (**a**), GO sheet (**b**), and GOPCL electrospun fibers (**c**) including their quantitative analysis of the myogenin-positive nuclei (**d**). Immunofluorescence staining of myosin heavy chain (MHC) (**e**–**g**) and the FESEM micrographs (**h**–**j**) of the related samples [204]. Reproduced with permission from Ref. [210]. Copyright 2021 the American Chemical Society.

Electrospinning technology plays an important role and is a fascinating approach for tissue engineering. Electrospinning technology contributes significantly to forming nanoscale or microscale fibrous structures possessing interjoined pores, which mimic the extracellular matrix (ECM) in living tissue [195]. This phenomenon is supported by observing the distinguished characteristics of skin as well as bones. Skin and bones are composed of extremely interconnected porous structures that are exploited in cell migration, a fundamental process in the development and continuation of multicellular organisms, and nutrient transport, important in transporting nutrients and chemical signals to the tissues and eliminating waste materials and heat [195,196,197]. The compact nanofibrous structures of electrospun scaffolds have significant and tuned porosity. The porosity decreases as the electrospun fiber diameter decreases from the micrometer to nanometer level, due to more close and compact packing. This phenomenon causes inferior cellular movement and generates a two-dimensional surface instead of the regular three-dimensional structure surrounding the ECM [198]. To date, electrospun nanofibrous matrices have been extensively explored in tissue engineering areas such as osseous tissue, muscle cells, chondrocytes, skin cells, neurons, and angiogenesis [195,199,200,201,202,203,204,205,206,207,208,209]. The addition of GO into the polymer matrix can change the physico-chemical properties of an electrospun nanofibrous scaffold utilized in tissue engineering. Oxygen-rich GO has hydrophilic features and, due to the relationship between GO and the electrospun nanofibrous matrices, the modified platform may exhibit increased hydrophilicity, cell growth, as evident in Figure 8, mesenchymal stem cell (MSC) osteogenic differentiation, mechanical strength, and biological activity, all essential parameters in tissue engineering [210]. GO has important applications in osteoregeneration with excellent outcomes [211], in which GO increases the stem cells located in the bone that play an important role in bone repair and growth [212]. This process is accomplished by Coulomb interactions and hydrophobic interactions with the proteins of the immediate small-scale environment of a plant cell or tissue [212].

**Figure 9:**
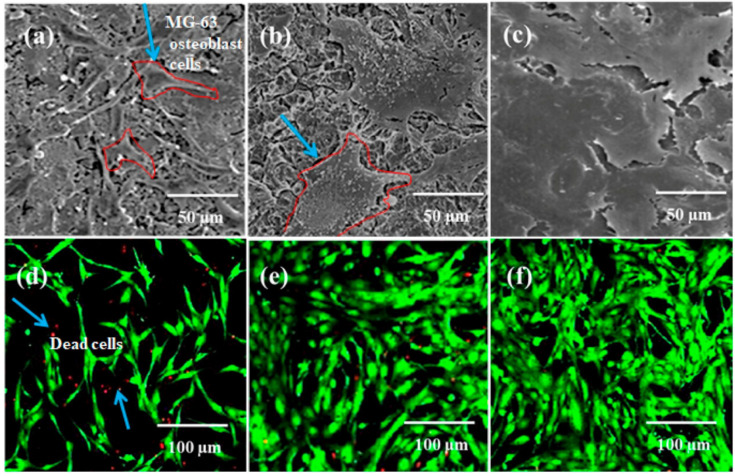
Analysis of cell adhesion and proliferation: (**a**–**c**) SEM pictures of BCp-PVp/GO 5 wt % electrospun composites after (**a**) 7 days, (**b**) 14 days, and (**c**) 21 days. (**d**–**f**) Live/dead analysis [212]. Reproduced with permission from Ref. [212]. Copyright 2018 MDPI.

Bone regeneration is one of the most common applications for electrospun GO nanofibrous matrices in tissue engineering based on the latest literature survey. The incorporation of GO enhanced the hydrophilicity of the electrospun nanofibrous matrices [213,214,215]. Biocompatibility of the electrospun nanofibrous matrices was shown to be unchanged in comparison with MG63 cells [216] but it was enhanced in different investigations utilizing human osteosarcoma cells (HOS), [217] MG-63 cells [218], bone marrow multipotent stem cells, medicinal signaling cells, or mesenchymal stem cells (BMSCs) [214], and C2C12 myoblast cells [219]. Cellular adhesion and proliferation were increased with the incorporation of GO in electrospun nanofibrous matrices [210,214,215,216,218,220,221,222,223,224]. Osteogenic expression and differentiation were significantly enhanced [214,217,218,225,226] in addition to mineral accumulation [220,222,223]. The enzymatic response time was increased, with respect to basic phosphatase [223,227]. The incorporation of GO in electrospun nanofibrous matrices enhanced mechanical properties significantly [216,217,218,220,222,223]. In one report, the elongation at break was enhanced by 462% and tensile strength increased by around 230%. The incorporation of GO in calcium phosphate and polyvinylpyrrolidone has found good application in biomedical implants [228]. These electrospun composite matrices exhibited excellent biocompatibility characteristics as was evident when MG-63 human osteoblast-like cells were added to calcium phosphate-polyvinylpyrrolidone/GO 5 wt % composites. Cell attachment and live/dead investigation exhibited no detrimental effects of the composite matrices (see Figure 9) [228].

**Figure 10:**
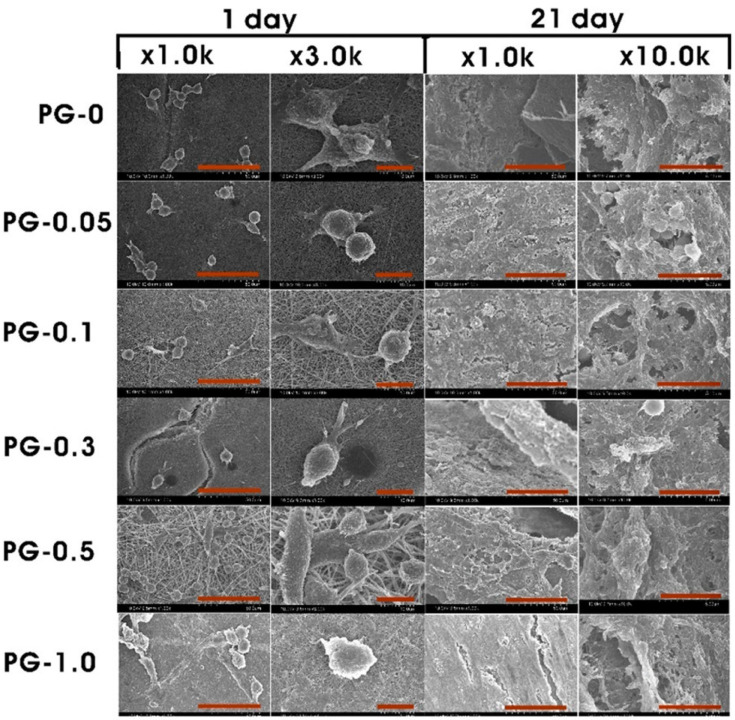
FE-SEM pictures of adhered and proliferated fibroblasts on PG scaffolds at magnifications of ×1.0k, ×3.0k, and ×10.0k; scale bars represent 50, 10, and 5 μm, respectively [210]. Reproduced with permission from Ref. [210]. Copyright 2021 the American Chemical Society.

This indicates that the above-mentioned material could be a potential applicant as scaffolding in osteoregeneration for biomedical engineering operations. In one report, poly (3-hydroxybutyrate-co-4-hydroxybutyrate/graphene oxide) (P34HB/GO) was used for osteoregeneration in laboratory rats with critical size congenital calvarial bone defects [229]. After testing, P34HB and P34HB with 1 mg/mL GO were analyzed for osteoregeneration. With two months of exposure, 47.2% and 60.77% fresh bone regeneration was seen in the P34HB and P34HB/GO groups, approximately [229]. These outcomes indicate the existence of GO in electrospun nanofibrous matrices and its positive impact on osteoregeneration and stimulation of bone formation by GO [229]. These electrospun nanofibrous matrices loaded with GO could be regarded as a medicinal alternative in tissue engineering, because of their porosity, biomechanics, osteoregeneration ability, and low-cost production process [229]. Another research team reported the utilization of electrospun silk fibroin scaffolds through the modification of GO with bone morphogenetic protein-2 (BMP-2) polypeptide for an enhanced osteoregeneration study [214]. NFs loaded with GO have been used in tissue engineering of muscle with diverse outcomes. PCL-GO nanofibrous composites have exhibited excellent biocompatibility for myoblast (C212) differentiation and have promising prospects in future-generation muscle tissue regeneration [219]. Nevertheless, these nanofibrous composites are also known for decreasing cell elongation by differentiation for randomly oriented and smooth fibers in CS12 cells [190] and therefore should be precisely evaluated when employing these scaffolds for tissue engineering of muscles [190]. Polyurethane (PU)/poly(ethylene oxide) (PEO)/GO electrospun nanofibrous matrices have exhibited degeneration of the scaffold, the absence of inflammation, and penetration of cells in vivo within the confines of the scaffold. PU/PEO/GO composite biomaterial has excellent biodegradability and biocompatibility and tuned porosity to perform as an appropriate material for regeneration of soft tissue in human beings [230]. Similarly, PU/PCL/GO electrospun nanofibrous composites exhibited reasonable compatibility with dermal fibroblasts, and the inclusion of GO enhanced the hydrophilicity of the polymeric matrices [231]. Electrospun poly(vinyl alcohol)/reduced graphene (PG) has been investigated for its promising prospects in engineering dermal tissue. Moreover, glucose-reduced graphene oxide (GRGO) was produced by using glucose as a reducing agent [232]. NFs were crosslinked by using acidic glutaraldehyde in a dimethyl ketone. PG nanofibrous matrices exhibited outstanding compatibility in the presence of CCD-986Sk (human fibroblast cell line), and significantly increased the metabolism after cell culture for three weeks compared to control groups, in the absence of GRGO, as shown in Figure 10 [232]. The incorporation of GO into a PVA matrix resulted in a minor shifting from hydrophilicity to hydrophobicity. However, in general, the PG nanofibrous matrices enhanced fibroblast cell growth and feasibility, exhibiting promising prospects of PG for dermal tissue engineering operations. Electrospun PCL/gelatin/GO nanofibrous composites exhibited great antibacterial qualities towards Gram-positive and Gram-negative bacterial strains. The nanofibrous composites may be employed as a scaffold of reasonable electrical conductivity in nerve regeneration, with drug release characteristics [184]. Drug release characteristics demonstrated pi stacking among TCH drug and GO and, therefore, enhanced the controlled delivery of TCH in relation to comparison groups in the absence of GO. Electrospun polycarbonate urethane (PCU)/GO nanofibrous matrices have been investigated in nerve regeneration applications [233]. Neural outgrowth of pheochromocytoma (PC 12) as a model cell line showed better results in the presence of the PCU/GO nanofibrous matrices compared to poly-L-lysine (PLL) material. Even though the mean diameter of neurites was identical in both GO- and PLL-coated surfaces, the neurites were elongated in the case of GO-coated matrices [233]. This work displays a unique surface engineering technology for GO coating on nanofibrous polymeric matrices. This unique platform could be employed as a 3D neuronic scaffold that can encourage neuroregeneration by providing better extracellular matrix surroundings [233]. Silk has been blended with GO to achieve an electrospinning technique and fabricate nanofibrous matrices for designing excitable nervous tissue, due to their ability to be electrically excited, which, subsequently, results in the production of action potentials [234]. Electrical conductivities were enhanced from approximately 4 × 10^−5^ S cm^−1^ in the dry state to 3 × 10^−4^ S cm^−1^ following the hydration process. Cellular adhesion and viabilities were found to be fine when these polymeric matrices were analyzed in the presence of neurinoma NG108-15 neuroblastoma cells. Progress in cell proliferation and metabolism was noticed for the GO-containing nanofibrous matrices, and subsequently increased more in the electroactive polymer composite of silk/rGO (demonstrating a modification in configurations when stimulated by an external electric field) [234]. Moreover, these electroactive polymeric matrices are promising candidates to enhance the nerve cellular response and can work as supporting material for nerve tissue regeneration applications.

### 3.3. Drug and Gene Delivery

**Figure 11:**
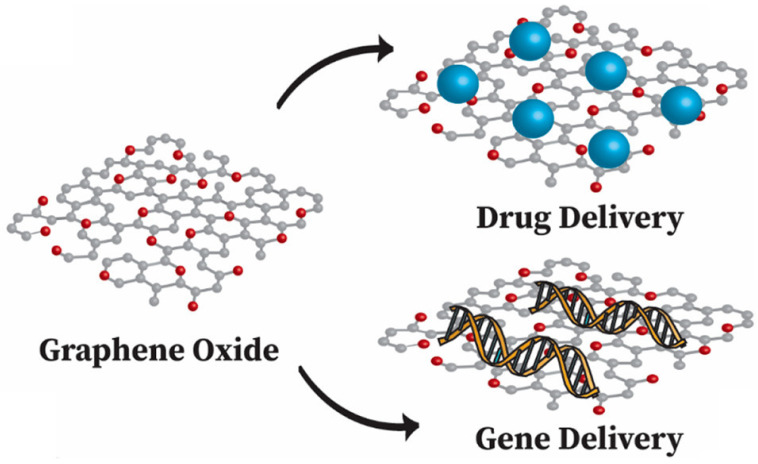
Schematic of drug and gene delivery from graphene oxide Reproduced with permission from Ref. [210]. Copyright 2021 the American Chemical Society.

Due to the excellent surface to volume ratio and stability, GO is extensively utilized in drug delivery systems and has promising prospects as a nanocarrier as shown in Figure 11 [235,236,237,238]. GO has demonstrated excellent biocompatibility and ion exchange characteristics, due to which GO is an exceptional applicant for drug delivery applications [236,239]. GO is known to load aromatic drugs with great productiveness by basic noncovalent interactions. The quinone part of aromatic drugs experiences pi stacking interactions due to the π conjugated system of GO, yielding hydrophobic characteristics [240]. The carboxyl and hydroxyl functional groups of GO permit powerful hydrogen bonding to occur, connecting the nanocomposites and aromatic drugs under study [240,241,242]. Oxygen-rich GO can be efficiently modified for addition of biomolecules, e.g., vitamin B9, which encourages and enhances drug loading capacity [243,244]. Integrating GO with polymers through electrospinning can have a positive impact and advantages in drug delivery operations. The solubility issues of hydrophobic drugs can be addressed by the use of appropriate polymers [245]. Defense against detrimental factors, such as environmental deterioration and disintegration, is provided by the use of suitable polymers [245]. A broad spectrum of polymers are used in NFs incorporating GO for drug delivery systems [233,236,240,245,246]. These drug-oriented polymers assist in developing the drug loading ability and increasing the delivery performance of the system. For example, poly (acrylic acid) (PAA) polymer has been reported to successfully transport and release ampicillin and cefepime drugs [247]. Similarly, poly(ε-caprolactone) (PCL) has been described as a transporting agent for dexamethasone and simvastatin drug delivery [236].

The mixture of polyvinylpyrrolidone (PVP) and PCL has been successfully involved in controlled delivery of vancomycin hydrochloride drug [233]. Zein, a versatile protein biopolymer, has been reported in precise delivery of tetracycline hydrochloride (TCH) and ketoprofen (KET) to treat infections caused by bacteria, such as pneumonia, other respiratory tract infections, and chronic wound [232,235]. Similarly, polymers such as poly(lactic) acid (PLA) have been investigated for their drug release properties, and biopolymers that include PEI and PLGA have been efficiently utilized for stable and transient transfection and immobilizing somatomedin C hormone [239,240].

**Figure 12:**
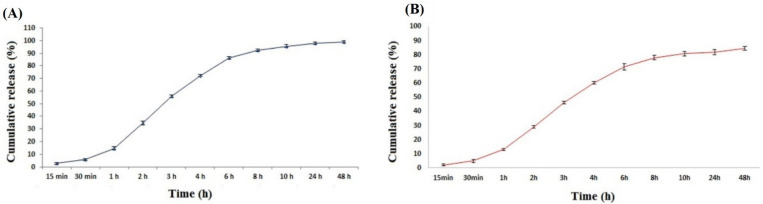
Release profiles of TCH in PVA/GT/TCH nanofiber (**A**) and PVA/GT/GO/TCH nanofiber (**B**) at pH 7.4. Reproduced with permission from ref [234]. Copyright 2020 Elsevier.

Quick/slow biphasic drug delivery systems have been investigated for quick release of a specific amount of drug for instant improvement of a patient’s condition, followed by sustained release, to escape periodic administration [248,249,250]. It has been reported that web thickness of the nanofibrous matrices has regulated drug delivery [249], alternatively, GO concentration controlled the drug release profile [250]. In general, GO incorporation has a positive impact on cumulative drug release and drug delivery rate was enhanced [251]. GO has also been consistently employed in electrospun nanofibrous matrices for antibacterial applications. rGO embedded polymeric nanofiber mats have been used for “on-demand” photothermally triggered antibiotic release of the antibacterial agents ampicillin and cefepime, which exhibited enhanced drug release, in relation to drug delivery at ambient temperature [245]. Both Gram-positive and Gram-negative bacterial strains were effectively suppressed by antibiotic drug delivery. An antibacterial wound dressing incorporating GO has exhibited extended drug release of tetracycline hydrochloride (TCH) [252]. Outstanding antibacterial characteristics were observed, and subsequently resulted in rapid wound healing [252]. It is well documented that GO concentrations up to 1% enhanced fibroblast cell growth and cell attachment [252]. Anti-inflammatory drugs, such as corticosteroids, and cholesterol have been included in the GO drug delivery process. Dexamethasone and Zocor medications have been utilized to enhance the number of osteoblasts, which arise from the osteogenic differentiation of MSCs [253]. Electrospun nanofibrous composites loaded with GO are promising candidates in neural cell treatment. The stimulation of autophagy in intracellular signal transduction pathways by methylene blue caused neural progenitor cell (NPC) proliferation on the electrospun nanofibrous composite to remain in the G0 phase [254]. NPCs were protected from programmed cell death, and tau phosphorylation proteins were reduced due to the influence of electrospun nanofibrous composites [254]. An innovative drug delivery method composed of polyvinyl alcohol and gum tragacanth blended with tetracycline accommodating GO and TCH has been reported for administration of active ingredients in transdermal operations [255]. The 3-(4,5-dimethylthiazol-2-yl)-2,5-diphenyl-2H-tetrazolium bromide (MTT) assay exhibited that the synthesized polyvinyl alcohol/gum tragacanth/graphene oxide or PVA/GT/GO/TCH composite NFs possess excellent cell growth when investigated in a usual endothelial cell line (HUVECs) [255]. This composite exhibited excellent antibacterial properties compared to control groups lacking GO or TCH. These excellent features are due to the existence of GO in electrospun nanofibrous composites and facilitated the controlled drug delivery and enhanced antibacterial characteristics as shown in Figure 12 [255]. Therefore, it is suggested that this composite material can be a potential applicant in precise and controlled drug delivery systems. Like drug delivery systems, gene delivery has been encouraged by the use of GO in electrospun nanofibrous composites. Genetic material (DNA) has been administered in stem cells to enhance transfection effectiveness and function as a medium for stem cell development and cell differentiation in biomedical engineering applications [256]. Similarly, somatomedin C has been delivered in electrospun GO/PGLA composites to increase neural stem cell (neurons, astrocytes, and oligodendrocytes) growth, differentiation, and continuation [257]. This electrospun composite approach exhibited exciting features for the development of the neuroprotective consequences of introduced nerves [257].

### 3.4. Cancer Therapy

**Figure 13:**
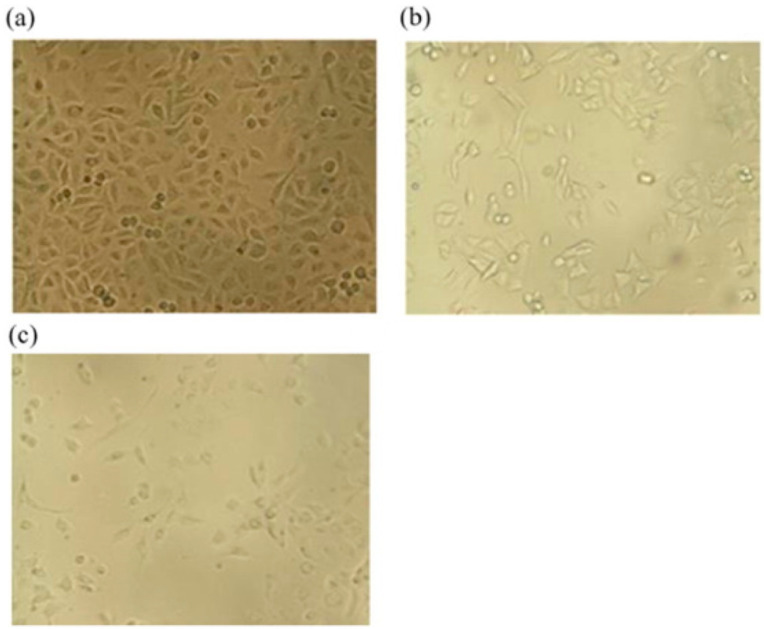
(**a**) Human lung cancer cells (A549), exposed to (**b**) pure DOX and (**c**) DOX-loaded PEO/CS/GO composite [237]. Reproduced with permission from ref [237]. Copyright 2015 Elsevier.

NFs containing GO and loaded with drugs have been investigated for their potential uses in cancer treatment. A polyethylene oxide/chitosan/graphene oxide (PEO/CS/GO) electrospun nanofibrous composite has been investigated for the controlled release of the chemotherapy drug doxorubicin (DOX) or adriamycin as shown in Figure 13 [258]. Drug loading of around 98% was achieved and the better prolonged controlled release was because of pi stacking relationships between GO and DOX in the loaded PEO/CS/GO electrospun nanofibrous composite [258]. Further investigations revealed the drug delivery dependence on different pH values. For instance, the fast drug release at pH 5.3 can be assigned to the chemical bonding between GO and the DOX drug, leading to an uncertainty in the presence of acidic surroundings. Cell viability investigations revealed that using human lung epithelial carcinoma (A549) has encouraging results when electrospun nanofibrous composites are utilized rather than free DOX in drug delivery studies. This would avert the damaging negative impact and effects of free DOX [258]. Moreover, more research was carried out on DOX drug delivery utilizing chitosan/poly(lactic acid)/graphene oxide/TiO_2_ (CS/PLA/GO/TiO_2_) electrospun nanofibrous composites to study the effect on human lung epithelial carcinoma (A549) [259]. First, sustained delivery of the DOX drug was observed and, subsequently, some uncontrolled release was observed when utilizing electrospun composites with 30–50 μm nanofiber diameters after 14 days of incubation time [259]. At pH 5.3, faster drug release of DOX was observed compared to pH 7.4, indicating a pH dependency as a result of a slight association between chemotherapy DOX drug and electrospun nanofibrous composites [258]. The existence of a magnetic field enhanced the prohibition of cell proliferation of the electrospun nanofibrous composites on human lung epithelial carcinoma cells [259].

It is well documented that adhesion features of biopolymers corresponding to cancer cells are associated with the control of the spreading of stage IV (4) cancer [260]. An association remains between biomaterials and the restriction of disease development [260]. In this regard, poly(caprolactone) (a biodegradable, biocompatible material)-based electrospun nanofibrous composite combined with GO results in the formation of PCLMF-GO nanofibrous composites and was reported for disease development restriction [261]. The covalent functionalization of GO and nitrogen plasma functionalization were employed for surface modification and to regulate physical and chemical characteristics of the electrospun nanofibrous composite to simultaneously encapsulate and eliminate the primary human dermal fibroblast adult cell line (HDFa) and human adenocarcinoma cells (MCF-7) [261]. Incorporation of GO has a positive impact and increased cell growth and attachment and directed cancer-associated fibroblast (CAF) encapsulation. Distinct photothermal therapy of the encapsulated cancer cells was significant and was feasible by high near-infrared absorbance (NIR) of GO [261]. This electrospun nanofibrous composite has a positive impact on the therapeutic effect of metastatic cancer cells and consequently decreased tumor distress and metastasis eruption in vivo. This electrospun nanofibrous composite also helps in prompt diagnosis of cancer cells in advance by employing an in vivo noninvasive fluorescent imaging technique [261]. An in vitro tumor prototype has been designed and developed based on graphene nanocomposites. In one study, GO was added to acetylated cellulose to compose nanocomposites for in vitro cell cancer examinations [262]. A human breast cancer cell line (MCF-7) was incorporated in the nanocomposite, and exhibited that cell development on these composites had a positive influence on cell viability, cell attachment, and proliferation in relation with those developed on basic CA [262]. Incorporation of GO also increased the mechanical strength of the nanocomposites. These outcomes suggest that GO performed an integral part in the human breast cancer cell study [262].

### 3.5. Wound Healing

**Figure 14:**
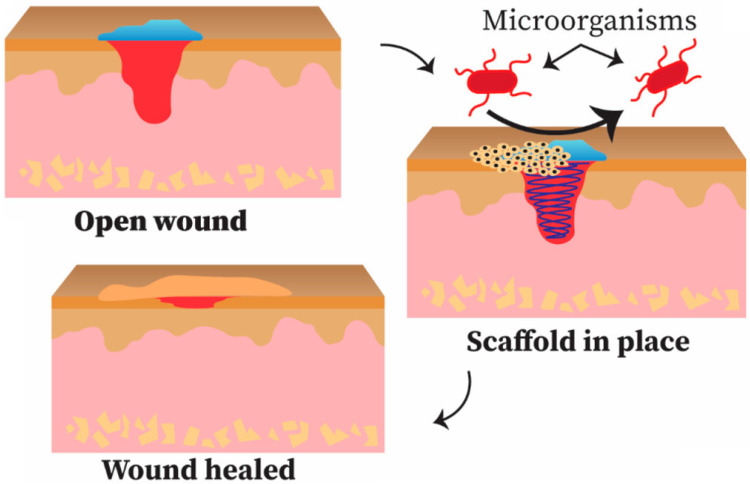
Schematic of how an electrospun scaffold containing GO/antimicrobial polymers may be effective in wound healing applications Reproduced with permission from Ref. [210]. Copyright 2021 the American Chemical Society.

Advancement and progress in nanotechnology and biomedical engineering introduce unique polymeric materials for wound healing and wound dressings. NFs have gained great interest for wound dressing and wound healing operations, due to their easier processing techniques. In general, electrospun fibers are on micrometer to nanometer scale and have a large surface to volume ratio, tunable porousness, and controlled morphology, that are all important parameters for drug loading and release [263,264,265,266]. Epithelialization (an essential component of wound healing) can be achieved, due to the close resemblance of the nanofibrous structure to extracellular matrix, and this encourages quick wound healing [267]. The proper selection of materials for the electrospinning technique is essential, and natural or synthetic polymers or their combination must be compatible with characteristics of the scaffold. Biopolymers are obtained from natural sources and are biocompatible, biodegradable, and harmless. Glycans, such as chitosan (a linear polysaccharide) and cellulose (structural polysaccharide), and proteins that include collagen (structural protein) and silk have been extensively used in electrospinning technology for drug release and wound dressing operations [268,269]. Most of these biopolymers have striking characteristics that facilitate wound healing. For instance, chitosan is an impressive antimicrobial agent because of its cationic properties [268] as shown in Figure 14.

**Figure 15:**
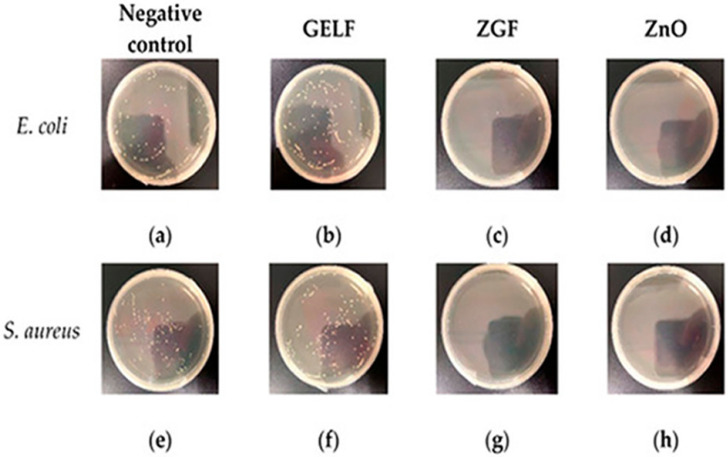
Effect of gelatin/zinc oxide/graphene oxide composite (ZnO) on *E. coli* and *S. aureus* compared to GELP and ZGF [266]. Reproduced under the Creative Commons Attribution License with permission from ref [258]. Copyright 2017 Elsevier.

There are multiple synthetic polymers being employed in wound dressing and healing, such as PVA, PLA, PEO, PVP, and PCL [270,271,272,273]. Generally, the mechanical strength of synthetic polymers is much better than natural polymers or biopolymers. These human-made polymers are efficiently dissolved in a broad spectrum of solvents, that facilitates the smooth functioning of the electrospinning technique [270]. In addition to excellent mechanical strength and hydrophilicity of GO, they also exhibit better antibacterial characteristics, due to the infiltration of the plasma membrane and the generation of very reactive oxygen radicals and the fact that GO is highly electrically conducting [274]. The blending of GO with suitable polymers employing the electrospinning technique can increase cell growth [275,276]. The electrospun nanofibrous matrices containing GO hold the moist air encircling the wound and speed up the healing process [56]. It is well documented that when GO is used, greater than optimum concentrations may have toxic effects and could have negative impacts and results in electrospun nanofibrous scaffolds used in wound dressing and healing applications [277]. Therefore, it is imperative to blend GO with suitable polymers having antimicrobial activity and biocompatibility to generate potent wound dressing and healing materials. The electrospun nanofibrous matrices containing GO have significant antibacterial and antimicrobial properties. These nanofibrous matrices have been generated with a range of polymers [278,279,280,281,282,283,284,285,286,287]. When GO is included in these electrospun nanofibrous matrices, Gram-positive and Gram-negative bacteria are killed [288] as shown in Figure 15. The influence of electrospun gelatin/zinc oxide graphene oxide (ZnO) nanofibrous composite on *E. coli* and *S. aureus* is shown in Figure 15 and these nanofibrous matrices were highly effective in killing both Gram-positive and Gram-negative bacterial strains.

**Figure 16:**
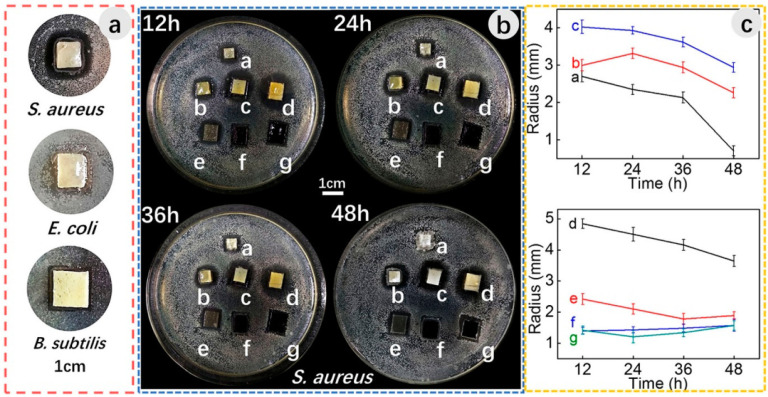
(**a**) Bacteriostasis of CS/PVA/GO0/Alli1 composites against various bacteria. (**b**) Bacteriostasis of distinctive composites against *S. aureus* over time. (**c**) The alteration of bacteriostatic circle diameter of various composites over time. Sample: a: CS/PVA/GO0/Alli0; b: CS/PVA/GO0/Alli0.5; c: CS/PVA/GO0/Alli1; d: CS/PVA/GO0/Alli2; e: CS/PVA/GO0.1/Alli2; f: CS/PVA/GO0.3/Alli2; g: CS/PVA/GO0.5/Alli2 [268]. Reproduced with permission from ref [268]. Copyright 2020 Elsevier.

The antimicrobial and antibacterial properties of electrospun nanofibrous matrices could be enhanced by the incorporation of antibiotic and antimicrobial drugs. Ciprofloxacin (Cip) and ciprofloxacin hydrochloride (CipHcl) are antibiotics that have been successfully integrated into electrospun GO nanofibrous matrices [284]. The nanofibrous architecture, the presence of GO within the NFs, and the nanofiber separation are essential for the drug incorporation and drug delivery. These electrospun nanofibrous matrices were highly effective at targeting and eliminating Gram-negative *E. coli*, Staphylococcus aureus, and Gram-positive B. subtilis [284]. The delivery profile of these electrospun nanofibrous matrices escaped the much anticipated “burst release”, and the extended delivery of two drugs takes place [284]. Cerium dioxide (CeO_2_) and peppermint oil (PM) incorporated electrospun GO nanofibrous matrices have also been investigated for their antibacterial characteristics [56]. It was reported that these composites showed much better antibacterial properties, because of the presence of the surface charge of cerium dioxide. The CeO_2_-PM oil-PEO/GO incorporated electrospun nanofibrous matrices were found to be less damaging to connective mouse tissue (L929) cells in relation to electrospun nanofibrous matrices composed of only cerium oxide and peppermint oil [56] and enhanced re-epithelization in wound healing [56,289]. Similarly, a wide range of drugs that include ibuprofen, ketoprofen, and vancomycin have been investigated and incorporated in a PCL/GO electrospun nanofibrous matrix for antimicrobial characteristics [289]. These electrospun nanofibrous composites used near-infrared light (NIR) for drug delivery for more than 72 h [289]. Similarly, allicin (the natural component of garlic) has been successfully incorporated into NFs composed of chitosan, PVA, and GO [290]. Furthermore, it was reported that the amount of GO in the electrospun nanofibrous matrices played an important role in controlled drug release of allicin. GO containing electrospun nanofibrous matrices acted as an excellent prolonged bacteriostatic agent as seen in Figure 16, in which the dimension of the bacteriostatic circle referring to the GO electrospun nanofibrous matrices does not shrink considerably in comparison to *S. aureus* [290]. Cytotoxicity investigation revealed that the collected electrospun nanofibrous matrices enhanced viability in relation to the L929 fibroblast cell line in the absence of GO. The inclusion of allicin did not suppress the cellular proliferation.

The electrospun nanofibrous matrices’ viability was reduced to marginally lower than 80% once GO was included, which implies that electrospun nanofibrous matrices carrying allicin as well as GO do not have any cytotoxic effect and can encourage cellular viability, because cellular viability above 70% is usually regarded as harmless in comparison to the biological materials under investigation [290]. The addition of allicin and GO as low as 0.1% into the electrospun nanofibrous matrices enhanced the cell–matrix adhesions or FAs and increased cellular growth [290]. Further studies showed that this material is a promising candidate and has good hydrophilic nature and reasonable water-holding capacity (WHC). The moist surface is helpful and facilitates wound healing [290]. The blending of GO and silver nanoparticles (AgNPs) was investigated for their antimicrobial properties [291]. A co-reduction method was employed to adhere AgNPs on the surface of the rGO.

Fabricated rGO-Ag was then uniformly distributed in PCL matrix and then subjected to an electrospinning technique to form smooth electrospun nanofibrous matrices. The incorporation of rGO-Ag increased the specific conductance, decreased the fiber diameter, and increased the mechanical properties [291].

**Figure 17:**
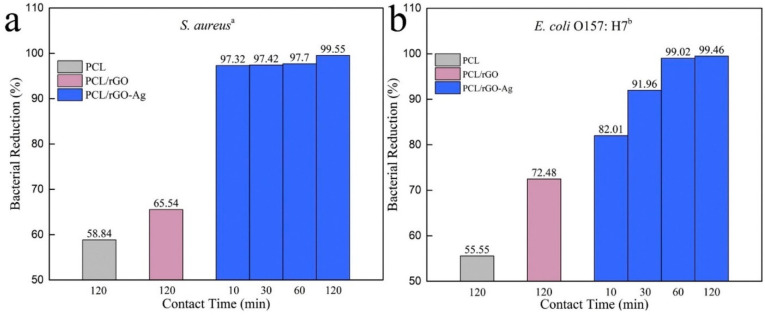
Antibacterial characteristics of PCL/rGO-Ag composites. (**a**) *S. aureus* inoculum was 7.00 × 10^5^ CFU/sample. (**b**) *E. coli* O157:H7 inoculum was 6.33 × 10^5^ CFU/sample [56]. Reproduced with permission from ref [56]. Copyright 2020 Elsevier.

Antibacterial properties of these electrospun nanofibrous matrices were around 99.55% and 99.46%, when tested on Staphylococcus aureus and Escherichia coli O157:H7 as shown in Figure 17, respectively, therefore rendering these nanofibrous matrices as appropriate material in wound healing applications [291]. Biocompatibility effects on human skin fibroblast cells [289,292], L929 cells (can be used in the development of novel anti-cancer treatments) [56,185], and MC3T3-E1 osteoprogenitor cells are well documented [281]. The fibroblast cells are of main interest due to their importance in wound healing applications [293]. It has been reported that electrospun nanofibrous matrices containing GO were innocuous to NIH/3 T3 fibroblast cells [293], enhanced cell growth of normal human dermal fibroblasts (NHDFs) [280], and encouraged cell attachment and activity of human dermal fibroblast cells (HDFs) [289]. A cytotoxic effect was observed when concentrations of GO of more than 1% were used [293], and this finding reveals that the fabricated nanomaterial should be blended with quercetin to enhance the electrospun nanofibrous matrices’ antibacterial properties [293]. The compatibility of a material with blood has also been reported when using GO-containing electrospun nanofibrous matrices, composed of PVA/GO [286] and collagen/carboxylated graphene [287]. The degradation investigations revealed that the gelatin/ZnO/GO electrospun nanofibrous matrices entirely degraded in a week, exhibiting well-controlled degradation properties [294]. At the same time, a separate study revealed that the impact of GO extended the degradation in simulated biological fluid (SBF), that indicates that electrospun nanofibrous matrices may be stable for extended duration in vivo tests [295]. The utilization of GO in electrospun nanofibrous matrices can be precisely controlled to expedite or decelerate the degradation rate and this is determined by the required applications [294,295]. Electrospun nanofibrous matrices containing GO are promising candidates for in vivo wound healing applications. Research on rat models has exhibited that electrospun nanofibrous matrices containing GO assisted in the regeneration of a substantial dermal injury, performing as a short-term dermal graft, two weeks after a post-operative procedure. The 1.5% GO concentration lead to compact and healthy skin regeneration, with wound healing of more than 99% recovery three weeks after a post-operative procedure [283]. One research team reported the re-epithelialization of wounds after two weeks in a rat model, in the presence of 1% GO in PVA/collagen/GO [285], and GO/Ag/arginine nanofibrous matrices [185]. Another study showed that electrospun nanofibrous matrices composed of PVA/GO had a significantly positive impact on wound healing and more than 90% efficiency was achieved at a 0.25% concentration of GO in rat models [286]. The incorporation of GO into electrospun nanofibrous matrices has increased mechanical properties significantly [185,280,286,287], and improved many physical and chemical properties [185,280,292] as reported by many research teams. Decisively, electrospun nanofibrous matrices containing GO have a broad range of applications. Characteristics that include antimicrobial properties, drug loading and drug delivery, tissue compatibility, hemocompatibility, re-epithelization, and outstanding mechanical features render these exciting materials very promising candidates for wound healing functions [288,289,292].

### 3.6. Biomaterials/Medical Equipment

**Figure 18:**
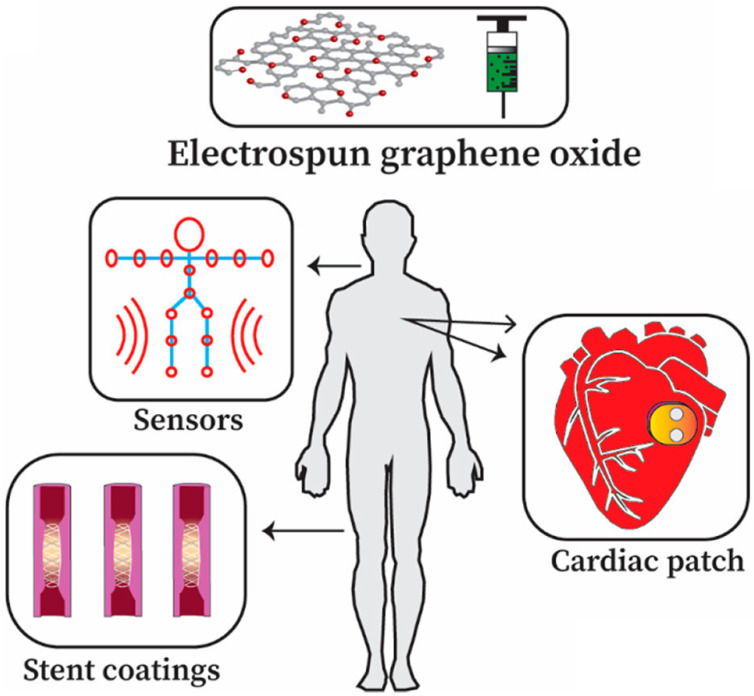
Potential for electrospun graphene oxide in biomaterials and medical device applications Reproduced with permission from Ref. [210]. Copyright 2021 the American Chemical Society.

Polymeric biomaterials have found tremendous importance in medical devices [296]. Surface modification of these medical devices is highly desirable to enrich biocompatibility and tissue–implant relationships [297]. Electrospun nanofibrous matrices are promising candidates and can be employed as biomaterials and medical equipment (see Figure 18). Electrospinning technology can be used to produce sub-micron diameter fiber scaffolding and utilized as artificial blood vessels/channels and coronary artery stents [298]. It has also tremendous importance in engineering electrically conductive cardiac patches [299,300]. These electrospun nanofibrous matrices can be designed in a way to utilize the exciting biological and mechanical features of the medical equipment [301]. Electrospun nanofibrous matrices encourage cellular intrusion and consequently lead to the deposition of ECM. This is because electrospun nanofibrous matrices perform as an ECM counterpart for distinct body cells, stem cells, and cancer cells to promote biomedical engineering, and aid in cellular proliferation, cellular specialization, and assembly modeling of cancer cells [302]. The incorporation of GO into nanofibrous matrices for use in medical equipment and specific biological materials can increase its hydrophilic nature, because of the presence of oxygen-rich functional groups on the material under study [152,303]. It is also well documented that incorporation of GO into electrospun polymeric material improves mechanical properties, including tensile strength, malleability, and specific conductance [286,299,304]. The addition of GO in small optimum concentrations in fields that include biomedical engineering, drug release, and wound dressing and healing is innocuous and may encourage cellular adhesion and cellular growth [210,222,230,249,250,252,281,283,285,295,298,305]. It is also well documented that incorporation of GO over particular optimum concentrations can lead to toxic effects, decreased cell activity, and production of free radicals [293]. Therefore, it is imperative to investigate the proper biomaterials and optimum GO concentration for a wide range of materials and their use in medical equipment. Electrospun nanofibrous matrices containing GO have been engineered and applied in electroactive cardiac patches. These engineered materials are appropriate as their electrical conductivity can be regulated to an extent that matches the natural cardiac electrical activity [299]. These features of this exciting material containing GO help in unfolding of fabric configurations in cardiomyocytes and human umbilical vein endothelial cells where the GO is close to the surface of the nanofibrous matrices [299]. Moreover, it has been found that electroactive cardiac patches containing GO play an essential secondary role as delivery vehicles for pharmaceuticals and biological molecules, and it was observed that heparin (an anticoagulant and used in preventing or in treatment of certain blood vessel, heart, and lung conditions) was effectively assimilated for the adsorption of bovine serum albumin [300]. This finding is outstanding because the functionalization of the nanofibrous matrices with heparin advances the biocompatibility of the medical equipment. Electrospun nanofibrous tubular grafts containing GO (0.5%) exhibited biological compatibility to cells derived from the endothelium of veins from the umbilical cord and mouse embryonic fibroblasts (3T3). These electrospun nanofibrous tubular grafts exhibited biocompatibility with artificial blood vessels concerning burst pressure and suture retention strength (the suture retention strength of synthetic vascular grafts is an important mechanical characteristic that affects the functioning of the vascular grafts). Moreover, it was shown that platelet attachment and human endothelial cell adhesion on the interior surface of the electrospun nanofibrous tubular graft were lower, thus exhibiting promising prospects in vascular tissue engineering [298]. In addition, electrospun nanofibrous matrices containing GO have been examined as a vascular stent coating [306]. The surface functionalization of the electrospun nanofibrous matrix with nitrogen-doped reduced graphene oxide (NrGO) generated an anion, which resulted in repelling of low-density lipoproteins (LDLs), also known as “bad” cholesterol, regarded as the fundamental reason for arteriosclerosis or atherosclerotic cardiovascular disease [306]. Similarly, the fabrication of GO/polycarbonate urethane (PU) films for implantable medical equipment was investigated [297]. The GO/PU films exhibited antibacterial properties for both Gram-positive and Gram-negative bacterial strains and continued low platelet adhesion (essential function in response to vascular injury) and biocompatibility in relation to mouse fibroblast L929 cells, thus rendering GO/PU films as promising material for the coating of cardiac implantable electronic devices [297]. The inclusion of GO increased the mechanical properties of the polyethylene terephthalate/GO electroconductive cardiac patch significantly. This material possesses the systematic and mechanical stability to establish electromagnetic coupling (EM) at myocardial infarction sites to maintain normal heart functioning [300] and enable diagnostics [307].

**Figure 19:**
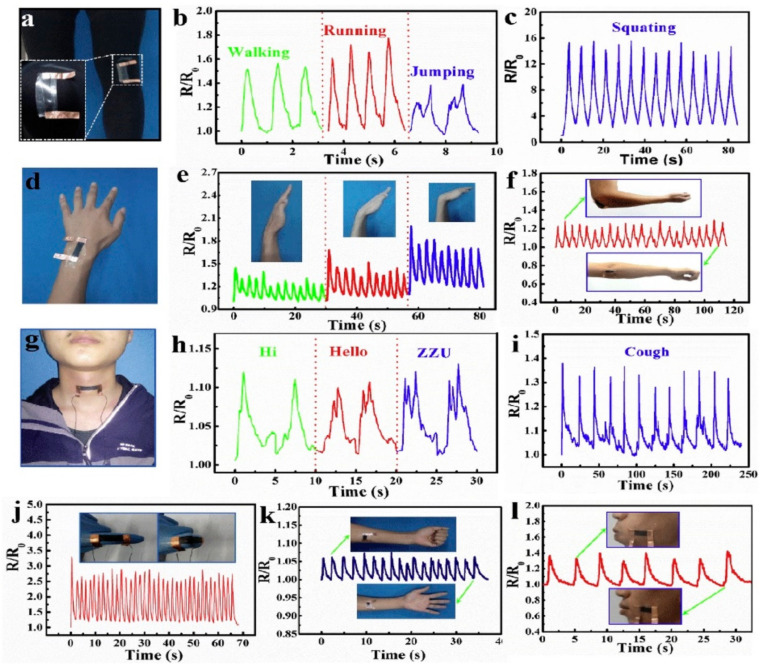
RGO/TPU strain sensors used to detect various human motions. (**a**) Picture of stocking with RGO/TPU strain sensor attached. (**b**,**c**) Curves of response under motions of walking, running, jumping, and squatting of RGO/TPU strain sensor on the knee. (**d**) Picture of RGO/TPU strain sensor on the wrist of a human. (**e**) Curves of response of diverse bending degrees of RGO/TPU strain sensor on the wrist. (**f**) Curves of response on the elbow under cyclic bending of RGO/TPU strain sensor. (**g**) Picture of RGO/TPU strain sensor attached on the throat. (**h**,**i**) Curves of response when the wearer coughs and says “Hi”, “Hello”, and “ZZU” (of RGO/TPU strain sensor). (**j**–**l**) Curves of response of RGO/TPU strain sensor on the finger, arm, and cheek. Reproduced with permission from Ref. [287]. Copyright 2016 the American Chemical Society.

Recent investigations have revealed that NFs containing GO can be used for applications in the electric power industry and sensing platforms. It has been shown that NFs containing GO can be used to fabricate a triboelectric nanogenerator (TENG) (new energy technology for converting human kinetic and ambient mechanical energy into electrical energy and works on the principle of Maxwell displacement current) [308]. The NFs containing GO were highly effective in the generation of electrical energy after submersion in phosphate buffer solution for four weeks. This device, called a nanogenerator, was employed to use electric energy to stimulate pheochromocytoma (PC12) cell lines, resulting in increased cellular adhesion and cellular growth [308]. More studies in future are required to evolve this platform as an environmentally friendly and economical technology for biomedical operations [308]. Influenced by this, an environmentally friendly, electrospun nanofibrous triboelectric nanogenerator composed of GO/PCL NFs and cellulose paper was investigated and turned out to be promising in biomedical engineering [308]. This material containing 4 wt% GO content showed good electrochemical properties. The nanofibrous structure had a positive impact on charge density accumulation [308]. The electrospun nanofibrous triboelectric nanogenerator composed of PCL loaded with 4 wt% GO with a dimension of 4 × 4 cm^2^ showed an increase up to 98% in relation to electromotive force (emf) or open-circuit voltage [308]. More than twenty LEDs were regularly operated by the triboelectric nanogenerator (TENG) configuration, by simple personal touch. The anionic charge from oxygen-rich GO and the nanopores from the electrospun nanofibrous matrices are the principal reason for this excellent performance [308]. Moreover, reports propose that, because of its harmless constituents, this GO-containing material could be regarded as a green energy producer, and address concerns of electric scraps in relation to self-driven medical instruments [308]. Electrospun nanofibrous matrices containing GO can generate a connection and improved 3D conductive network due to the better interaction of NFs with one another and have significant importance in motion sensing applications [309]. Extraordinary electronic properties, ductility, stability, and sensitiveness render this GO-loaded material composition a promising applicant in human motion sensing as shown in Figure 19. The wide extent of motion sensing that includes normal human activities such as walking, hopping, finger and muscle movements, speaking, sneezing, etc. indicates that GO-loaded NFs are promising candidates as an advanced wearable gadget and may have substantial importance in health/fitness tracking applications [309]. State-of-the-art research has investigated the use of NFs containing GO in biosensing platforms. For this purpose, GO/PVA electrospun nanofiber scaffolds have been reported. The copper (Cu) nanoflower has been developed and fabricated and has important application as a glucose biosensor based on electrochemical measurements as shown in Figure 15 [307]. The modified gold chip displayed a lower limit of quantification of 0.018 μM for glucose. These findings suggest that this material system can be a promising candidate in electroanalysis of glucose in biological fluid for mobile testing and rapid diagnostics.

Quantum chemical modeling established on the density functional theory (DFT) can be applied to investigate the interface characteristics of graphene surfaces. The utilization of DFT-established quantum chemical models to characterize the graphene surface provides numerous benefits, such as the capability to investigate the surface at the atomic level. Contemporary analytical techniques, which include high-resolution transition electron microscopy and scanning tunneling microscopy, can be utilized to investigate the graphene surface at the molecular level. However, these techniques are expensive and have certain limitations. Consequently, quantum chemical modeling is needed to investigate the surface on an atomic level. In addition to atomic-scale investigation, conceptual DFT-established quantum chemical models (Fukui functions and dual descriptors are extremely useful in realizing electron transfer (ET) reactions and yield important input into surface energy levels. Fukui functions are chemical descriptors that are beneficial to analyze the reactivity of systems toward electron transfer.

## 4. Conclusions

The use of electrospun graphene oxide for biosensing and biomedical applications has great potential. Electrospinning technology provides the polymer NFs with controlled pore geometries, preferable dimensions, and chemical functionalities which are useful for the production of novel nanostructure materials with biosensing and biomedical capabilities [170]. It is reported that GO above certain optimum concentrations can lead to loss in feasibility and biocompatibility [192,293,310,311]. In addition, there is potential to revise and functionalize NFs on a mass scale, permitting this technology to deliver a broad range of sensing activities, in relation to other techniques, primarily due to the large surface area, better porosity, and control of the chemical compositions [312]. NFs can be functionalized by assimilating GNMs during electrospinning or a post-electrospinning process onto the surface of the as-prepared NFs to increase the important qualities for fabricating electrochemical biosensors. The development and the hydrophilic nature of GNMs make it a potential applicant for several medical-based operations [179,210,253,313,314]. For instance, when GO has been incorporated into NFs to fabricate a tissue-engineered scaffold, the cell adhesion, growth, and general biocompatibility of the scaffold have been enhanced [210,220,222,223]. Due to the large specific surface area and high porosity, electrospun nanofibers furnish immobilization sites and can attach to bioreceptors, permitting bioreceptor–analyte interactions and increasing the current response for sensing the model biological molecules. The large specific surface area and high drug and gene loading ability, by hydrogen bonding, produce GO-containing electrospun composites convenient for drug delivery applications [236,238,249,250,252]. Conclusively, electrospinning of GNMs for biosensing and biomedical applications has been proven to be advantageous. Controlling the GNM concentrations within the electrospun nanofiber composite will invalidate any unsatisfactory result, permitting the development of outstanding sensing, biocompatibility, and biomaterials.

### Perspective and Future Outlook

Electrospinning has emerged as an essential technology to generate functional NFs with the required structure and configurations. Nevertheless, several key issues inhibit the transfer of electrospinning technology from the laboratory to commercial business, that include spinneret configuration, rheological parameters, concentration of solution under investigation, electric field strength and charge distribution, atmospheric pressure and temperature, volume flowrate, needle to collector distance, and collector structure and orientations. These factors have a critical impact on the reproducibility of NF production and at various places. Moreover, the incorporation of GNMs into electrospun polymeric NFs using electrospinning technology has been regarded as an outstanding approach to generate effective sensing materials exploiting the benefits of the excellent and structural properties of GNMs and versatile electrospun polymeric NFs. However, to achieve top-quality electrochemical biosensors, certain issues need to be addressed that include increasing GNM concentration without accumulation or aggregation and how to expand the area of immobilization active zones for biomolecules. Generally, the optimization of the interactions between graphene and its composites in GNMs is very important to enhance the electrocatalytic performance of electrochemical biosensors. The pre- and post-processing procedures are suitable modification techniques for the production of GNMs and electrospun polymeric NFs for biosensors employing electrospinning technology. The pre-processing technique works on the principle of mixing GNMs with the polymer pre-electrospinning process and is an effective way to generate NFs containing GNM nanomaterials for biosensing applications with great physico-chemical characteristics, reproducibility, and extended stability. Similarly, the post-processing technique works on the principle of superficially coating or furnishing GNMs on the NFs prepared in advance for prompt interaction with biological molecules and, subsequently, results in improved efficiency of electrochemical biosensors. The pre-processing technique exhibits outstanding ability for biosensing platforms, but the requirements of this technique are rough conditions such as aggressive stirring, precise development of GNMs, and the utilization of complex instruments such as co-axial electrospinning. Further difficulties with the pre-processing technique include the distribution, geometry, orientation, and the suitable loading of GNMs into the polymeric matrix. According to additional guidelines, more investigations are needed to regulate the combined effort and result of GNMs and their relationship with polymeric nanocomposites in the course of the electrospinning technique to enable the homogeneity and proper distribution of graphene-based materials. The post-processing procedure normally has a better capability of employing GNMs promptly for biosensing operations, because of the potential to furnish a huge surface area of NFs prepared in advance with GNMs, therefore enlarging the prospects of an interface between GNMs and biological molecules to promote precise detection of biological samples. The considerable issue of the post-processing technique is its capability to create precise relationships between the GNMs and the polymeric nanofiber matrices, because GNMs cannot be conveniently assimilated with the NFs prepared in advance. Hence, more investigations are needed for the optimization of the coating techniques of GNMs on the surface of the NFs to enhance the intermolecular forces connecting GNMs and the electrospun polymeric NFs. One research team [315] described a productive approach to enhance the integration and stability of CVD-produced graphene and electrospun polyacrylonitrile NFs by means of a heat treatment technique to manufacture a transparent sensor with much better electrical conductivity, mechanical properties, analytic sensitivity, and stability. Even though NFs containing GO have been extensively investigated in tissue engineering/regenerative medicine, drug delivery and gene delivery, wound healing/dressing, and medical equipment applications, there is a substantial desire for state-of-the-art technology to reach new horizons of GO utilization in biomedical applications. Development in these areas is still in the initial phase. Although the growth in this area has promising prospects, some issues remain with regard to utilizing NFs containing GO for biomedical operations. The lack of understanding and education of GO−cell relationships requires full attention and investigation before the GO–electrospun nanofiber composites can be categorized as biocompatible material. The cellular uptake phenomenon with respect to GO requires proper investigation, understanding, and continuous improvement [316,317]. The harmful effects of GO inside and outside living cells need to be addressed. It is well documented that GO above a certain specific concentration is of safety concern [293,310]. There are only a few publications in which the toxic nature of GO has been considerably investigated [309,310,311,312,313,314,315]. The flat shape and surface electrical (negative) charges of GO are directly linked to cell cytotoxicity and apoptosis [315]. The mechanism of cytotoxicity is associated with the elevated intracellular levels of reactive oxygen species and damage of plasma membranes. Furthermore, toxicity with respect to GO’s impact has been associated with incubation state and classification of cells [316]. Some studies have revealed that inhaling GO nanomaterials leads to pulmonary infection. Additionally, it has been investigated that exposure to GO in mouse models leads to pulmonary fibrotic scarring in the lung tissue after three weeks of impact [317]. GO taken orally can become deposited in the abdomen and colon of mice. Denudation and death of mucosal epithelial cells from the stomach to the small intestine occurred when GO was orally given to mice on a daily basis during lactation [316]. Moreover, full-scale and well-organized investigations are required to evaluate the possible safety issues and toxicity of utilizing GO for biomedical operations. This phenomenon would involve the proper investigation of various chemical synthetic approaches of GO, tissue compatibility, bioabsorbability, and toxic nature of the nanomaterial under study. With reference to additional guidelines, the NFs containing GO have promising prospects in tissue engineering, because of their capability for encouraging cell attachment, cell growth, differentiation, and cell activity. GO has been found to effectively promote essential signals and growth factors for much better tissue culture [317]. In addition to applications such as drug release, NFs containing GO could be developed as delivery vehicles of proteins, growth factors (effective in stimulating cell growth, wound healing/dressing, and cell differentiation), and important biological molecules to help in disease diagnostics such as chemotherapy. Similarly, NFs containing GO have significant applications in wound healing operations. The additional guidelines suggest that this material should be developed to enhance actions toward operational environments by integrating this material with other substances with known medical use [317]. The utilization of NFs containing GO in medical equipment is a comparatively unique and innovative sector with promising prospects. According to most recent investigation, there could be significant improvements in the field of sensing platforms (such as motion sensing and biosensing) [307,309]. Furthermore, NFs containing GO have substantial importance in the designing of TENGs for biomedical engineering [308,317].

This review highlights the most recent accomplishments in electrospun nanofiber construction of functional nanostructures to lead to novel materials with improved properties for biosensing and biomedical applications by utilizing the outstanding characteristics of GNMs. It is worth mentioning that the proper investigation and alteration of GNMs and their surface functionalization such as reduction of GO to rGO or inclusion of additives enhance their distribution within the polymeric matrix, result in increasing specific conductance, and enhance the ability of the polymeric material to resist the action of heat, electrochemical, and mechanical characteristics of the electrospun nanofibrous matrices. In addition, the precise optimization of electrospinning parameters results in the generation of porous NFs, core–shell structures, and hollow nanostructures which enhances the surface to volume ratio and, subsequently, increases the immobilization active sites for the biological molecules under study. This review features the contemporary advancement of graphene-based materials, the outstanding functioning of GNMs to manufacture future-generation biosensing and biomedical devices, and the significance of electrospinning technology and designs of electrospun nanofibrous composites in relation to bridging the gap between laboratory facilities and industry.

## Data Availability

Data underlying the results presented in this paper are available from the corresponding author upon reasonable request.
